# Safety of age-dosed, single low-dose primaquine in children with glucose-6-phosphate dehydrogenase deficiency who are infected with *Plasmodium falciparum* in Uganda and the Democratic Republic of the Congo: a randomised, double-blind, placebo-controlled, non-inferiority trial

**DOI:** 10.1016/S1473-3099(22)00658-2

**Published:** 2022-11-30

**Authors:** Walter R Taylor, Peter Olupot-Olupot, Marie A Onyamboko, Pimnara Peerawaranun, Winifred Weere, Cate Namayanja, Peter Onyas, Harriet Titin, Joy Baseke, Rita Muhindo, Daddy K Kayembe, Pauline O Ndjowo, Benjamin B Basara, Georgette S Bongo, Charles B Okalebo, Grace Abongo, Sophie Uyoga, Thomas N Williams, Chiraporn Taya, Mehul Dhorda, Joel Tarning, Arjen M Dondorp, Naomi Waithira, Caterina Fanello, Kathryn Maitland, Mavuto Mukaka, Nicholas J P Day

**Affiliations:** Mahidol Oxford Tropical Medicine Clinical Research Unit, Mahidol University, Bangkok, Thailand; Centre for Tropical Medicine and Global Health, Nuffield Department of Medicine, University of Oxford, Oxford, UK; Mbale Clinical Research Institute, Mbale, Uganda; Department of Public Health, Busitema University, Mbale, Uganda; Kinshasa School of Public Health, University of Kinshasa, Kinshasa, Democratic Republic of the Congo; Mahidol Oxford Tropical Medicine Clinical Research Unit, Mahidol University, Bangkok, Thailand; Mbale Clinical Research Institute, Mbale, Uganda; Kinshasa School of Public Health, University of Kinshasa, Kinshasa, Democratic Republic of the Congo; Mbale Clinical Research Institute, Mbale, Uganda; KEMRI-Wellcome Trust Research Programme, Kilifi, Kenya; KEMRI-Wellcome Trust Research Programme, Kilifi, Kenya; Institute of Global Health Innovation, Imperial College London, London, UK; Mahidol Oxford Tropical Medicine Clinical Research Unit, Mahidol University, Bangkok, Thailand; Mahidol Oxford Tropical Medicine Clinical Research Unit, Mahidol University, Bangkok, Thailand; Centre for Tropical Medicine and Global Health, Nuffield Department of Medicine, University of Oxford, COxford, UK; Mahidol Oxford Tropical Medicine Clinical Research Unit, Mahidol University, Bangkok, Thailand; KEMRI-Wellcome Trust Research Programme, Kilifi, Kenya; Institute of Global Health Innovation, Imperial College London, London, UK; Mahidol Oxford Tropical Medicine Clinical Research Unit, Mahidol University, Bangkok, Thailand; Centre for Tropical Medicine and Global Health, Nuffield Department of Medicine, University of Oxford, Oxford, UK

## Abstract

**Background:**

WHO recommends gametocytocidal, single low-dose primaquine for blocking the transmission of *Plasmodium falciparum*; however, safety concerns have hampered the implementation of this strategy in sub-Saharan Africa. We aimed to investigate the safety of age-dosed, single low-dose primaquine in children from Uganda and the Democratic Republic of the Congo.

**Methods:**

We conducted this randomised, double-blind, placebo-controlled, non-inferiority trial at the Mbale Regional Referral Hospital, Mbale, Uganda, and the Kinshasa Mahidol Oxford Research Unit, Kinshasa, Democratic Republic of the Congo. Children aged between 6 months and 11 years with acute uncomplicated *P falciparum* infection and haemoglobin concentrations of at least 6 g/dL were enrolled. Patients were excluded if they had a comorbid illness requiring inpatient treatment, were taking haemolysing drugs for glucose-6-phosphate dehydrogenase (G6PD) deficiency, were allergic to the study drugs, or were enrolled in another clinical trial. G6PD status was defined by genotyping for the *G6PD* c.202T allele, the cause of the G6PD-deficient A− variant. Participants were randomly assigned (1:1) to receive single low-dose primaquine combined with either artemether–lumefantrine or dihydroartemisinin–piperaquine, dosed by bodyweight. Randomisation was stratified by age and G6PD status. The primary endpoint was the development of profound (haemoglobin <4 g/dL) or severe (haemoglobin <5 g/dL) anaemia with severity features, within 21 days of treatment. Analysis was by intention to treat. The sample size assumed an incidence of 1·5% in the placebo group and a 3% non-inferiority margin. The trial is registered at ISRCTN, 11594437, and is closed to new participants.

**Findings:**

Participants were recruited at the Mbale Regional Referral Hospital between Dec 18, 2017, and Oct 7, 2019, and at the Kinshasa Mahidol Oxford Research Unit between July 17, 2017, and Oct 5, 2019. 4620 patients were assessed for eligibility. 3483 participants were excluded, most owing to negative rapid diagnostic test or negative malaria slide (n=2982). 1137 children with a median age of 5 years were enrolled and randomly assigned (286 to the artemether–lumefantrine plus single low-dose primaquine group, 286 to the artemether–lumefantrine plus placebo group, 283 to the dihydroartemisinin–piperaquine plus single low-dose primaquine group, and 282 to the dihydroartemisinin–piperaquine plus placebo group). Genotyping of *G6PD* identified 239 G6PD-c.202T hemizygous males and 45 G6PD-c.202T homozygous females (defining the G6PD-deficient group), 119 heterozygous females, 418 G6PD-c.202C normal males and 299 G6PD-c.202C normal females (defining the non-G6PD-deficient group), and 17 children of unknown status. 67 patients were lost to follow-up and four patients withdrew during the study—these numbers were similar between groups. No participants developed profound anaemia and three developed severe anaemia: from the G6PD-deficient group, none (0%) of 133 patients who received placebo and one (0·66%) of 151 patients who received primaquine (difference −0·66%, 95% CI −1·96 to 0·63; p=0·35); and from the non-G6PD-deficient group, one (0·23%) of 430 patients who received placebo and one (0·25%) of 407 patients who received primaquine (−0·014%, −0·68 to 0·65; p=0·97).

**Interpretation:**

Gametocytocidal, age-dosed, single low-dose primaquine was well tolerated in children from Uganda and the Democratic Republic of the Congo who were infected with *P falciparum*, and the safety profile of this treatment was similar to that of the placebo. These data support the wider implementation of single low-dose primaquine in Africa.

**Funding:**

UK Government Department for International Development, UK Medical Research Council, UK National Institute for Health Research, and the Wellcome Trust Joint Global Health Trials Scheme.

## Introduction

Facing the growing threat of artemisinin-resistant *Plasmodium falciparum* in the low-transmission Greater Mekong Subregion of southeast Asia, in 2021 WHO recommended the addition of single low-dose primaquine to artemisinin-based combination treatments to counter artemisinin-resistant *P falciparum*.^1^ Evidence suggested that a dose of 0·25 mg/kg bodyweight, a third of the original 0·75 mg/kg dose, would kill mature gametocytes and block transmission to mosquitoes.

The main safety concern with primaquine is dose-dependent acute haemolysis in individuals with glucose-6-phospate dehydrogenase (G6PD) deficiency. G6PD is an essential enzyme for the replenishment of reduced glutathione to counter oxidative stress in red blood cells and other tissues caused by drugs and infections.^2^ G6PD deficiency is an X-linked recessive disorder with more than 140 gene mutations, which results in G6PD-deficient variants with different degrees of enzyme deficiency.^2^ Hemizygous males and homozygous females are most severely affected, whereas heterozygous females have a mixed population of G6PD-normal and G6PD-deficient red blood cells, resulting in a broad range of enzyme activities and variable risk of oxidant-induced haemolysis.

G6PD deficiency in sub-Saharan Africa is mostly caused by the c.202C→T mutation in the *G6PD* gene (known as the A− variant), with reported allele frequencies of around 2% in The Gambia, around 17% in Kenya, and around 21% in Nigeria.^3^ The A− variant causes relatively mild G6PD deficiency compared with other variants,^2^ and is associated with residual enzyme activity of around 20% in hemizygous males and homozygous females and around 40% in heterozygous females, compared with the median activity of a G6PD-normal population.^4^ More severe variants reported in Africa include G6PD Santa Maria (approximately 7% prevalence in The Gambia) and G6PD Mediterranean (up to around 4% prevalence in Sudan).^3^

*P falciparum* infection itself causes acute haemolysis. In children treated for *P falciparum* infection in sub-Saharan Africa, mean haemoglobin concentrations declined to nadir on day 2, then increased to stabilise after 4–6 weeks.^5^ Severe anaemia warranting a blood transfusion can develop soon after treatment,^6^ and occurred in 10 (1·5%) of 679 children treated with an artemisinin combination therapy in the Democratic Republic of the Congo.^7^ A meta-analysis reported anaemia or severe anaemia as serious adverse events in 10 (0·2%) of 5948 patients.^8^

The WHO-recommended single low-dose primaquine target dose of 0·25 mg/kg is now supported by several studies of transmission blocking efficacy and gametocyte carriage that use a range of primaquine doses.^9–12^ Safety studies in patients with G6PD deficiency who are infected with *P falciparum*, asymptomatic individuals who are carriers of *P falciparum*, and healthy volunteers across the age spectrum all support the recommendation of not needing to test for G6PD deficiency.^11,13–15^ The overall, absolute, and fractional declines in haemoglobin concentrations overlap among patients with or without G6PD deficiency and those who are treated with single low-dose primaquine or artemisinin combination therapy, with declines of up to 4 g/dL, or a more than 25% decrease from the baseline. Mean differences in the decrease of haemoglobin concentrations between patients with and without G6PD deficiency have generally been small (around 1 g/dL).^11,13,14^ Marked differences in the decrease of haemoglobin concen-trations according to *G6PD* genotype were shown clearly by Shekalaghe and colleagues,^16^ who used much higher doses of primaquine (ranging from 0·75 mg/kg to more than 1·0 mg/kg).

Although the safety evidence base for the use of single low-dose primaquine has increased, a fundamental weakness is the paucity of data regarding individuals in the key risk group for primaquine haemolysis: children younger than 5 years from sub-Saharan Africa with falciparum malaria; G6PD deficiency; and other inherited haemoglobin disorders such as sickle cell trait, sickle cell disease, and α-thalassaemia. To close this knowledge gap, we conducted a study of single low-dose primaquine in children with uncomplicated falciparum malaria in Uganda and the Democratic Republic of the Congo.

The Mbale Regional Referral Hospital (MRRH) is in an area of perennial malaria transmission with peaks during the rainy seasons in March to May and August to October. The estimated annual entomological inoculation rate in Tororo district, 45 km from Mbale, is 125 (95% CI 72–183).^17^ MRRH admits between 20 000 and 22 000 children per year with 65% testing positive for *P falciparum*. Local prevalence rates of sickle cell disease are 1%, sickle cell trait 15%, homozygous α^+^-thalassaemia 6·6%, and heterozygous α^+^-thalassaemia 37·5%.^18^ The only G6PD-deficiency variant identified is the *G6PD* c.202T A− variant; 12·7% of males are hemizygotic and 8·6% of females are homozygotic (Williams TN, unpublished). These haemoglobinopathy rates are similar to those from Jinja (east of Kampala) but lower than rates in Tororo (eastern Uganda) and higher than those in Kanungu (western Uganda).^19^

The Kinshasa Mahidol Oxford Research Unit (KIMORU) covers the health zones of Kingasani (urban) and Maluku I (urban–rural), where malaria is hyperendemic, perennial, and affects predominantly children younger than 5 years. One study reported country-wide prevalence rates of 1·4% for sickle cell disease and 16·9% for sickle cell trait in 31 204 neonates.^20^ The prevalence of α^+^-thalassaemia in the general Kinshasa population is unknown.

## Methods

### Study design and participants

This randomised, double-blind, placebo-controlled, non-inferiority, safety trial of single low-dose primaquine combined with either open-label artemether–lumefantrine or dihydroartemisinin–piperaquine took place at MRRH, Mbale, eastern Uganda and KIMORU, Kinshasa, Democratic Republic of the Congo.

Children who were potentially eligible for the study were identified by a nurse or clinician at MRRH or KIMORU. A rapid structured assessment was performed to exclude children with symptoms or signs indicating the need for hospitalisation. Children were enrolled if they were aged between 6 months and 11 years and presented with clinically uncomplicated disease, a measured fever (≥37·5°C aural) or a history of fever for 72 hours or less, and either a positive malaria slide for *P falciparum* (mono or mixed infection) of any parasitaemia in Kinshasa or a positive rapid diagnostic test (SD Bioline Malaria Ag Pf/Pan test, SD BioLine, Suwon, South Korea) in Uganda.

Exclusion criteria were clinical danger signs or any sign of severe malaria (eg, inability to take or retain fluids or oral medications, confusion, prostration, convulsions, respiratory distress, or passing of red or cola-coloured urine [known as blackwater fever]); haemoglobin concentration less than 6 g/dL; comorbid illness requiring inpatient treatment (physician’s judgement); concurrent use of drugs known to cause haemolysis in G6PD deficiency (eg, dapsone or nalidixic acid); known allergy to primaquine, artemether–lumefantrine, or dihydroartemisinin–piperaquine; or previous enrolment into the current trial or co-enrolment into any other clinical trial.

The study was approved by the Oxford University Tropical Research Ethics Committee (reference 53-16), the Mbale Regional Hospital Institutional Review Committee (MRRH-REC OUT – COM 006/2017), the National Drug Authority (CTA0028), and the Uganda National Council for Science and Technology (HS2205), and in the Democratic Republic of the Congo by the Ministry of Higher and University Education, the University of Kinshasa Public Health School Ethics Committee, and the city of Kinshasa Provincial Government Health Minister (ref 135/MIN.SAN. AFFSOC&ACHUM/CM/JD/2017). Oral consent was obtained from the parents or guardians of the patients to screen for malaria, G6PD deficiency, and haemoglobin concentrations. Written informed consent was obtained from all legal guardians of eligible patients.

### Randomisation and masking

Participants were randomly assigned to one of four groups: artemether–lumefantrine plus single low-dose primaquine; artemether–lumefantrine plus placebo; dihydroartemisinin–piperaquine plus single low-dose primaquine; or dihydroartemisinin–piperaquine plus placebo. Computer-generated randomisation lists with a seed for reproducibility were generated for each site, G6PD group, and age-dosing band (appendix p 3). The lists were provided by the trial statistician (MM) to the packaging company (Bilcare Research, Pune, India) to prepare the blister packs and opaque envelopes.

Four labelled blister packs were prepared for the four age groups of the age-based regimen: A (6–11 months), B (1–5 years), C (6–9 years), and D (10–11 years). Identical (in terms of size and dark brown coating) primaquine and placebo tablets were produced by Centurion Laboratories (Vadodara, India). Blisters A and B contained 2·5 mg of primaquine or placebo, blister C contained 5 mg, and blister D contained 7·5 mg. Blister packs and envelopes were labelled with sequential numbers (randomisation number), starting with 001, and D (for deficient) or N (for normal) to denote G6PD status (appendix p 4). The reverse of each blister card was labelled with the artemisinin combination therapy to be given, as per the randomisation list (appendix p 5). In KIMORU, all study drugs were crushed and dissolved in 2 mL of water and milk before administration using a teaspoon. Similarly, for children younger than 5 years in MRRH, study drugs were dissolved in water and then administered with milk. Older children were given whole tablets to swallow and encouraged to drink milk or take a small snack. In both sites, the 2·5 mg tablet was halved using a pill cutter and administered as stated.

Patients were recruited according to the sequence of the blister pack numbers (ie, randomisation number) for each G6PD group and age-dosing band. All site investigators and teams were completely masked to the allocation of primaquine or placebo. At the end of the study, the statistician checked the randomisation of the blister packs against the randomisation list to verify that the procedure was done as planned.

### Procedures

Oral artemether–lumefantrine (Coartem; Novartis, Basel, Switzerland) and dihydroartemisinin–piperaquine (Fosan Pharma, Guilin, China) were given to participants in doses determined by weight according to the manufacturers’ instructions and WHO recommendations. One tablet of artemether–lumefantrine contains 20 mg artemether and 120 mg lumefantrine and was given by one of four weight groups (5 kg to less than 15 kg, 1 tablet; 15 kg to less than 25 kg, 2 tablets; 25 kg to less than 35 kg, 3 tablets; and 35 kg or more, 4 tablets). Dihydroartemisinin–piperaquine was given by one of eight weight groups (5 kg to less than 8 kg, 20 mg dihydroartemisinin plus 160 mg piperaquine; 8 kg to less than 11 kg, 30 mg plus 240 mg; 11 kg to less than 17 kg, 40 mg plus 320 mg; 17 kg to less than 25 kg, 60 mg plus 480 mg; 25 kg to less than 36 kg, 80 mg plus 640 mg; 36 kg to less than 60 kg, 120 mg plus 960 mg; 60 kg to less than 80 kg, 160 mg plus 1280 mg; and 80 kg or more, 200 mg plus 1600 mg). Primaquine was dosed by age, using a newly proposed regimen (6 months to less than 1 year, 1·25 mg; 1−5 years, 2·5 mg; 6−9 years, 5 mg; 10–14 years, 7·5 mg; and 15 years or older, 15 mg).^3^

Research nurses administered all study drugs. Children were encouraged to eat a small snack or drink milk to reduce the risk of primaquine-induced abdominal pain and vomiting and to increase the absorption of lumefantrine and piperaquine. Patients were observed for 60 min after administration. Children who vomited within 30 min of drug administration were re-treated with the same dose of artemether–lumefantrine or dihydroartemisinin–piperaquine and primaquine or placebo. Half doses were given to children who vomited 31–60 min after drug administration. Patients with persistent vomiting were treated with parenteral artesunate, according to local guidelines.

The following assessments were carried out on enrolled patients before drug administration: history (current illness, medical and drug history, family medical histories, and symptoms checklist); physical examination; methaemoglobin (upgraded Masimo Rad57 oximeter; Masimo International, Neuchâtel, Switzerland) and haemoglobin (HemoCue; Angelholm, Sweden) concentrations and haematocrit (at KIMORU only); blood films for malaria and reticulocyte count (new methylene blue-stained thin film); blood spots for parasite genotype and molecular markers of resistance; and venous blood collection for full blood count (DxH500, Beckman Coulter [MRRH], Indianapolis, IN, USA, and quantitative buffy coat analysis [KIMORU]), liver function and creatinine concentration in a subset of patients (COBAS C111, Roche [MRRH], Basel, Switzerland, FUJI DRI-CHEM 4000i, Fujifilm, Tokyo, Japan [KIMORU]), and genotyping for G6PD deficiency, sickle cell trait and sickle cell disease, and α^+^-thalassaemia. During follow-up, some investigations were repeated (appendix p 2). For the analysis of haemoglobin concentrations over time, we used values obtained from the HemoCue measurement system.

Giemsa-stained, thick and thin blood films were read (magnification of 1000 ×) independently by two micro-scopists and mean parasitaemias were calculated. If more than 200 parasites per 10 fields were counted on a thick blood film, parasitaemia was subsequently measured on a thin blood film; 2000 red blood cells were assessed and the number of parasitised red blood cells was noted. Below this threshold, the number of parasites per 500 white blood cells on the thick film were counted. A negative slide was declared after reading at least 200 high-power microscopy fields. Asexual and sexual parasitaemias (gametocytes) were calculated on a thin film as (number of parasites per 2000 red blood cells × haematocrit × 125·6)/2. For patients in whom haematocrit was not measured, values were calculated as 5·62 + (2·60×haemoglobin concentration).^21^ On the thick film, parasitaemia was measured as total number of counted parasites × 16, assuming a total white cell count of 8000/μL.

The G6PD rapid diagnostic test was used at screening to track the number of recruited patients who were G6PD deficient. The G6PD deficiency A– variant, defined by the *G6PD* c.202T allele, was characterised from genomic DNA using the Agena MassArray IPLEX platform, as previously described.^22^ Genotyping for sickle cell disease and the common form of α^+^-thalassaemia in Africa, which is caused by a 3·7-kbp deletion in the α-globin gene, was conducted by PCR.^23^ Patients who were heterozygous for the β^S^ mutation in *HBB* were classed as having sickle cell trait and patients who were homozygous for this mutation were classed as having sickle cell disease. Patients with a single α-globulin deletion were classed as heterozygous for α^+^-thalassaemia and those with two α-globin deletions were classed as homozygous for α+-thalassaemia.

A symptom checklist, comprising common adverse events and symptoms of anaemia, was administered at baseline, on days 3 and 7, and then weekly to day 42. Vital signs (pulse, blood pressure, respiratory rate, and aural or axillary temperature) were recorded every 12 h in hospital and at each outpatient visit. We used International Council for Harmonisation definitions of adverse and serious adverse events, which were recorded from after the first dose of study drug to the last follow-up day, treated as clinically indicated, and observed until resolution or stabilisation. For grading of adverse events, we used the 2007 National Institutes of Health Division of Microbiology and Infectious Diseases Pediatric Toxicity Table.

For this study, we reclassed increases in serum creatinine as treatment-emergent acute kidney injury using the Kidney Disease: Improving Global Outcomes (KDIGO) guidelines: baseline ×1·5−1·9 (grade 1), baseline ×2·0−2·9 (grade 2), and baseline × 3·0 or more (grade 3).^24^ The adverse events were classed as unrelated, unlikely to be related, possibly related, probably related, or definitely related to the study drugs.

### Outcomes

The primary outcome was the development of either profound anaemia (haemoglobin <4 g/dL) or severe anaemia (haemoglobin <5 g/dL) and clinical signs of severity (eg, respiratory distress, impaired consciousness, prostration, or coma) within 21 days of starting treatment.

The secondary outcomes were: mean haemoglobin concentrations over time; day of mean nadir haemoglobin concentration; maximal absolute and fractional (percentage) decreases in haemoglobin concentration (nadir concentration ≤14 days *vs* baseline); proportions of children with fractional decreases in haemoglobin concentration of at least 30%; proportions of children with haemoglobin recovery (day 42 haemoglobin concentration greater than day 0 haemoglobin concentration); mean reticulocyte counts over time; *G6PD*, sickle cell, and α+-thalassaemia genotypes; changes in the concentration of methaemoglobin over time; clinical and laboratory adverse events; and gametocyte carriage over time. Factors explaining haemoglobin dynamics and profound or severe anaemia, primaquine pharmacokinetics, cytochrome P450 2D6 polymorphisms, parasite clearance kinetics, and day 42 cure rates will be reported elsewhere.

### Statistical analysis

The sample size was chosen on the basis of showing non-inferiority of artemisinin combination therapy plus single low-dose primaquine compared with artemisinin combination therapy plus placebo in the development of the primary outcome in the G6PD-deficient group, defined as a genotypically-confirmed hemizygous male or homozygous female. A previous study at KIMORU reported a 1·5% blood transfusion rate for decompensated anaemia in patients with uncomplicated *P falciparum* malaria who were treated with artemisinin combination therapy.^7^

Assuming a primary outcome rate of 1·5% in the artemisinin combination therapy plus placebo group, a non-inferiority margin of 2·5%, a one-sided alpha of 0·025, a power of 80%, and a 5% loss to follow-up, the sample size was calculated as 393 per group. We sought to recruit 800 G6PD-deficient and 800 non-G6PD-deficient patients (ie, genotypically heterozygous females and genotypically normal males and females). Owing to challenges in recruitment, it was agreed by the trial funder (UK Medical Research Council) and steering committee that the non-inferiority margin should be increased to 3%, resulting in a sample size of 280 patients per group (560 in the G6PD-deficient group and 560 in the non-G6PD group).

Data were collected on paper case report forms, checked with the source documents, and entered onto MACRO (version 4.9.1), a web-based, good clinical practice compliant, clinical management data system. After cleaning, data were analysed with Stata (version 17), following a prospectively written statistical analysis plan.

Analysis of the primary outcome was by intention to treat, whereby patients were analysed according to the group of randomisation, irrespective of the treatment that was actually given. We used all available data within the first 21 days to compute the primary outcome. Additional safety outcomes were also assessed in all randomly assigned patients, of whom all received at least one dose of the study drugs.

Non-inferiority of artemisinin combination therapy plus single low-dose primaquine in patients with G6PD deficiency was determined by calculating the risk difference between the artemisinin combination therapy plus single low-dose primaquine and artemisinin combination therapy plus placebo groups with two-sided 95% CIs. A lower bound of the 95% CI of the risk difference (placebo group – primaquine group) of −3% or higher confirms non-inferiority of the artemisinin combination therapy plus single low-dose primaquine treatment.

Categorical data were analysed by Fisher’s exact test. Continuous data between two groups were analysed by an unpaired *t* test, and continuous data between three groups were analysed by one-way ANOVA, transforming the data as needed. Multivariable regression assessed factors for the initial decline in haemoglobin concentration (analyses of other haemoglobin parameters will be reported elsewhere). All tests were two-sided and a p value of <0·05 denoted statistical significance. The statistical analysis plan was finalised and signed before database lock and the breaking of the code.

An independent Drug Monitoring and Ethics Committee was established to review trial safety data and receive data on haemoglobin concentrations in real time when there was a decrease from the baseline value of 30% or more, or when haemoglobin concentrations were less than 5 g/dL.

The trial is registered at ISRCTN, number 11594437.

### Role of the funding source

The funders had no role in study design, data collection, data analysis, data interpretation, writing of the report, or decision to publish.

## Results

**Figure 1:**
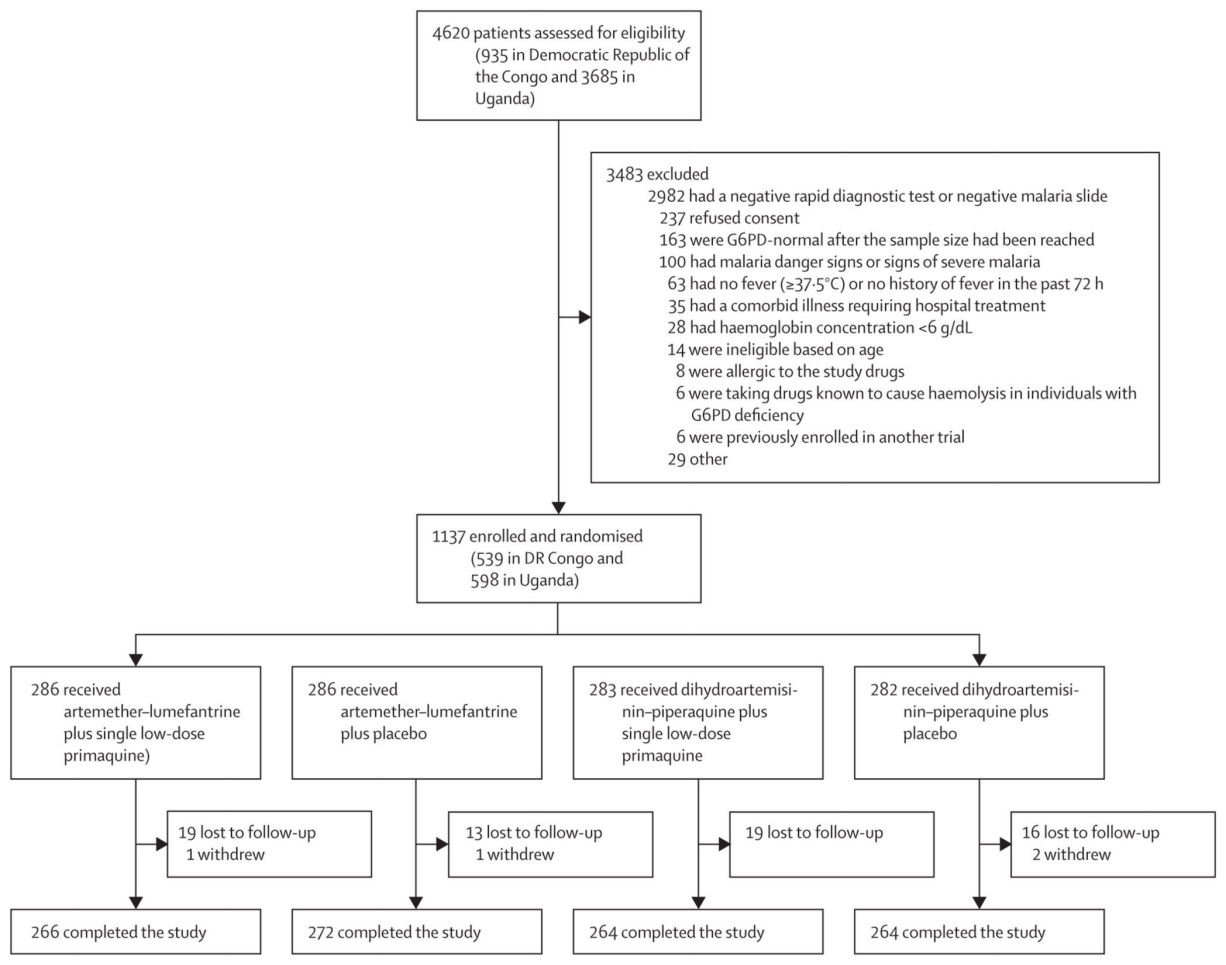
Trial profile G6PD=glucose-6-phosphate dehydrogenase.

Participants were recruited at KIMORU between July 17, 2017, and Oct 5, 2019, and at MRRH between Dec 18, 2017, and Oct 7, 2019. 4620 patients were screened (935 at KIMORU and 3685 at MRRH), 3483 patients were excluded, and 1137 were recruited (539 at KIMORU and 598 at MRRH). The most common reasons for exclusion were not having a malaria infection (n=2982) and declining to participate in the study (n=237); only 28 patients were excluded for having a haemoglobin concentration of less than 6 g/dL (figure 1, appendix pp 7–8). All participants who were randomly assigned to study groups received study drugs, and 71 (6·2%) of the 1137 participants were lost to follow-up or withdrew from the study. Of the 1137 participants who were recruited and randomly assigned to study groups, 1066 completed the study. 286 patients were assigned to the artemether–lumefantrine plus single low-dose primaquine group; 286 to the artemether–lumefantrine plus placebo group; 283 to the dihydroartemisinin–piperaquine plus single low-dose primaquine group; and 282 to the dihydroartemisinin–piperaquine plus placebo group.

Across all randomised groups, the median age of participants was 5 years and children had been unwell for a median of 2 days (table 1, appendix p 9–11). 1129 (99·2%) of 1137 children were of normal nutritional status, 292 (25·7%) of 1136 (one patient did not have vital sign data) had a fever at presentation, and physical signs were unremarkable apart from a splenomegaly rate per group of 20–25%. Mean haemoglobin concentrations were 10·5–10·7 g/dL across all participants and 9·9–10·1 g/dL in children younger than 5 years. Moderate anaemia (haemoglobin <8 g/dL) was present in only 76 (6·7%) of 1137 patients.

*G6PD* genotyping identified 239 *G6PD* c.202T hemizygous boys and 45 *G6PD* c.202T homozygous girls (classed as G6PD deficient); 119 heterozygous girls; 418 *G6PD* c.202C hemizygous boys and 299 *G6PD* c.202C homozygous girls (classed as non-G6PD deficient); and 17 children of unknown genotypic status. Mean baseline haemoglobin concentration by genotypic class was 10·3 g/dL (SD 1·6) in the G6PD-deficient group, 10·5 g/dL (1·7) in heterozygous girls, and 10·7 g/dL (1·6) in the G6PD-normal group; only the difference between the G6PD-deficient and G6PD-normal groups was significant (p=0·0002, Bonferroni-adjusted). 585 (52·4%) of 1116 (some had missing data) participants had either heterozygous or homozygous α+-thalassaemia and only three children had sickle cell disease. 151 (25%) of 598 patients recruited at MRRH had positive rapid diagnostic test results but were malaria-slide negative. Overall, 201 (18%) of 1136 (one patient either had no slide done or their slide was not readable) patients had gametocytaemia.

Only three (0·3%) of 1137 children met the prospectively defined primary outcome; all had haemoglobin concentrations of less than 5 g/dL with features of severe malaria. One of these patients was in the G6PD-deficient group (0 [0%] of 133 receiving artemisinin combination therapy plus placebo and one [0·66%] of 151 receiving artemisinin combination therapy plus single low-dose primaquine [difference −0·66, 95% CI −1·96 to 0·63; p=0·35, appendix p 13]); and two of these were in the non-G6PD-deficient group (one [0·23%] of 430 receiving artemisinin combination therapy plus placebo and one [0·25%] of 407 receiving artemisinin combination therapy plus single low-dose primaquine [−0·014%, −0·68 to 0·65; p=0·97]).

**Figure 2:**
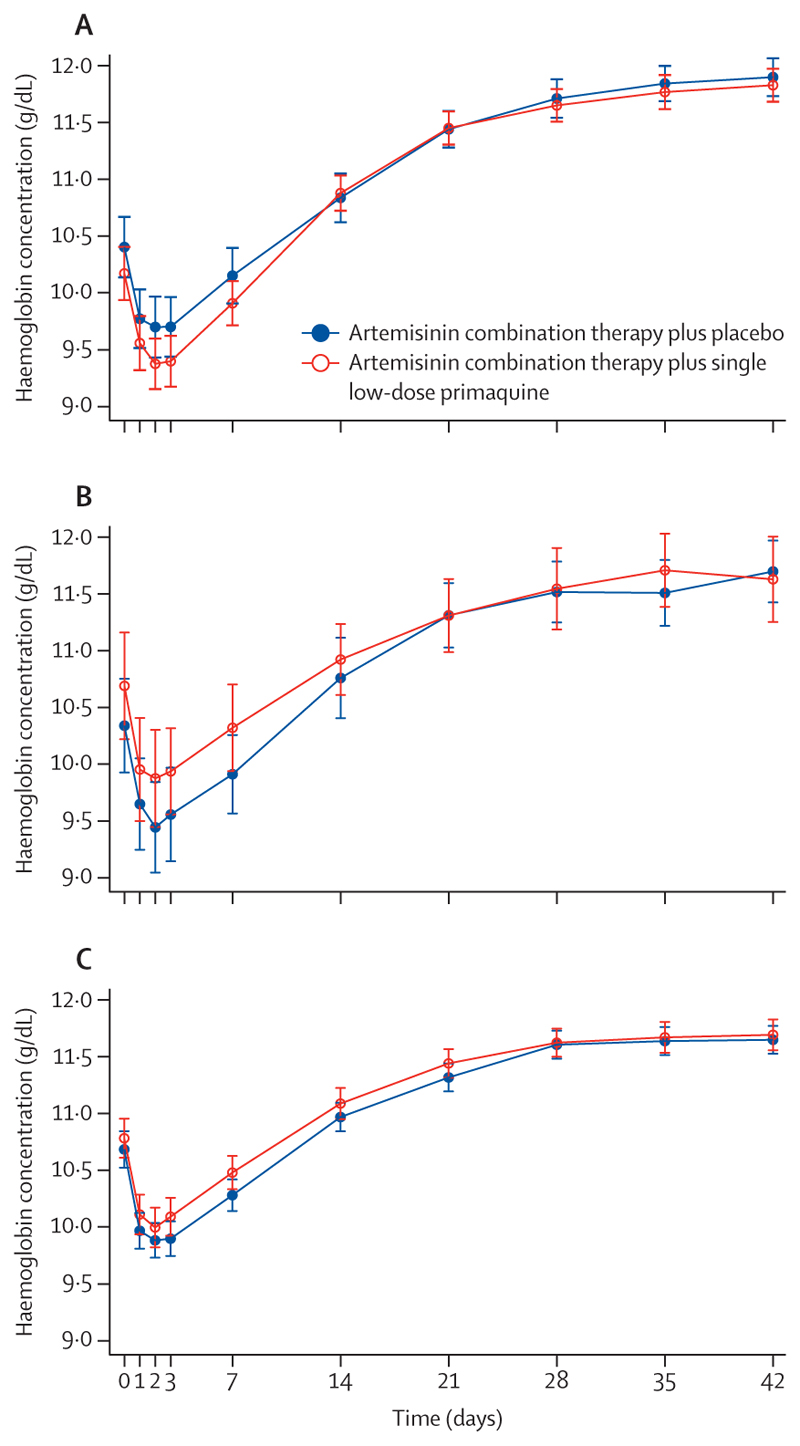
Mean haemoglobin concentrations over time in patients treated with artemisinin combination therapy plus either primaquine or placebo (A) *G6PD* c.202T hemizygous boys and *G6PD* c.202T homozygous girls (G6PD-deficient). (B) *G6PD* heterozygous girls. (C) *G6PD* c.202C hemizygous boys and *G6PD*-c.202C homozygous girls (G6PD-normal). Data were collected over the 42-day follow-up period of the study, after treatment with artemisinin combination therapy plus placebo or artemisinin combination therapy plus single low-dose primaquine. Error bars indicate 95% CI. G6PD=glucose-6-phosphate dehydrogenase dehydrogenase.

For all patients, the mean haemoglobin concentration decreased to nadir on day 2 (mean day 3 concentrations were similar), recovering thereafter (figure 2).

Within the first 14 days of follow-up, the maximum absolute and fractional decreases in haemoglobin concentrations were 4·7 g/dL and 40% in the G6PD-deficient group, 5·0 g/dL and 37% in the heterozygous group, and 7·1 g/dL and 58% in the G6PD-normal group (appendix p 14). The proportions of children with fractional decreases of 30% or more ranged from 3% to 5% among the different groups. The only significant difference between the primaquine and the placebo groups was in the median fractional decrease of haemoglobin concentration (11·1% primaquine *vs* 9·0% placebo) in the G6PD-deficient group, but this significance was lost in a multivariable analysis (appendix p 15).

Factors associated with a larger fractional reduction in haemoglobin concentration were a higher parasite count and longer history of illness, whereas increasing age, splenomegaly, and sickle cell trait or sickle cell disease were associated with smaller fractional decreases (ie, were protective against declines in haemoglobin). G6PD status, haemoglobin type (normal, thalassaemia trait, or thalassaemia carrier), dose of primaquine, nutritional status, hepatomegaly, and sex were not significant explanatory variables (appendix p 15).

By day 42, haemoglobin recovery was seen in 421 (74·5%) of 565 children in the artemisinin combination therapy plus single low-dose primaquine group and 423 (75%) of 564 children in the artemisinin combination therapy plus placebo group (p=0·89).

For all patients combined, an initial decrease in mean reticulocyte count was observed, followed by an increase that peaked on day 7 to stabilise by day 28 (appendix p 16). The trends by G6PD status were the same (data not shown).

Substantial overlap in methaemoglobin concentrations was seen in all G6PD groups at baseline and after treatment (appendix p 17). Median methaemoglobin values tended to be higher in the artemisinin combination therapy plus single low-dose primaquine groups, but the differences were small. The maximum recorded methaemoglobin values in patients who received artemisinin combination therapy plus single low-dose primaquine were 3·7% in G6PD-deficient patients, 3·4% in heterozygous patients, and 7·2% in G6PD-normal patients; corresponding values in the artemisinin combination therapy plus placebo group were 3·2% in G6PD-deficient patients, 2·7% in heterozygous patients, and 3·7% in G6PD-normal patients. Of patients in the G6PD-normal group who were treated with single low-dose primaquine, one of 717 had a day 0 methaemoglobin concentration of 7% at baseline; however, this value was an outlier, and subsequent values were lower (5·3% on day 4 and 2·5% on day 14). Analysis of methaemoglobin concentrations according to G6PD status showed no significant differences between groups (appendix pp 18–19).

Single low-dose primaquine was well tolerated. On day 0, 40 (3·5%) of 1137 children had early vomiting (defined as within 1 hr of treatment) and they were similarly distributed across the treatment groups, as were those with mild or moderate abdominal pain, nausea, diarrhoea, and headache (table 2). No children reported shortness of breath or difficulty breathing. For all adverse events, comparisons between artemether–lumefantrine treatment and dihydroartemisinin–piperaquine treatment (data not shown) and between artemisinin combination therapy plus single low-dose primaquine and artemisinin combination therapy plus placebo were not statistically significant.

Median and IQR alanine transaminase and aspartate transaminase concentrations remained within normal limits over time, with small downward trends seen over time; downward trends in the median total bilirubin and creatinine concentrations were more marked. All comparisons between the artemisinin combination therapy plus single low-dose primaquine and artemisinin combination therapy plus placebo groups were not significant (appendix pp 20–21). A few children had grade 3 or 4 neutropenia, thrombocytopenia, transaminitis, or hyperbilirubinaemia, and all grades of acute kidney injury (table 2).

16 patients had 17 doctor-reported serious adverse events: 11 patients received blood transfusions for anaemia, three patients had intercurrent illnesses (tetanus, diabetes, or severe malaria with pneumonia), one child had a grade 4 increase in aspartate transaminase (474 IU/L) and alanine transaminase (668 IU/L), and one child had a Division of Microbiology and Infectious Diseases grade 4 (KDIGO grade 3) increase in serum creatinine concentration (day 0, 0·2 mg/dL; day 7, 1·7 mg/dL). Of the 11 children who received blood transfusions (six from the artemisinin combination therapy plus single low-dose primaquine group and five from the artemisinin combination therapy plus placebo group), ten received transfusions in the first week and one on day 31. Four of the children who received transfusions were G6PD deficient, two of whom received artemisinin combination therapy plus single low-dose primaquine; of the seven children who received transfusions in the non-G6PD-deficient group, four received artemisinin combination therapy plus placebo (appendix p 22).

**Figure 3:**
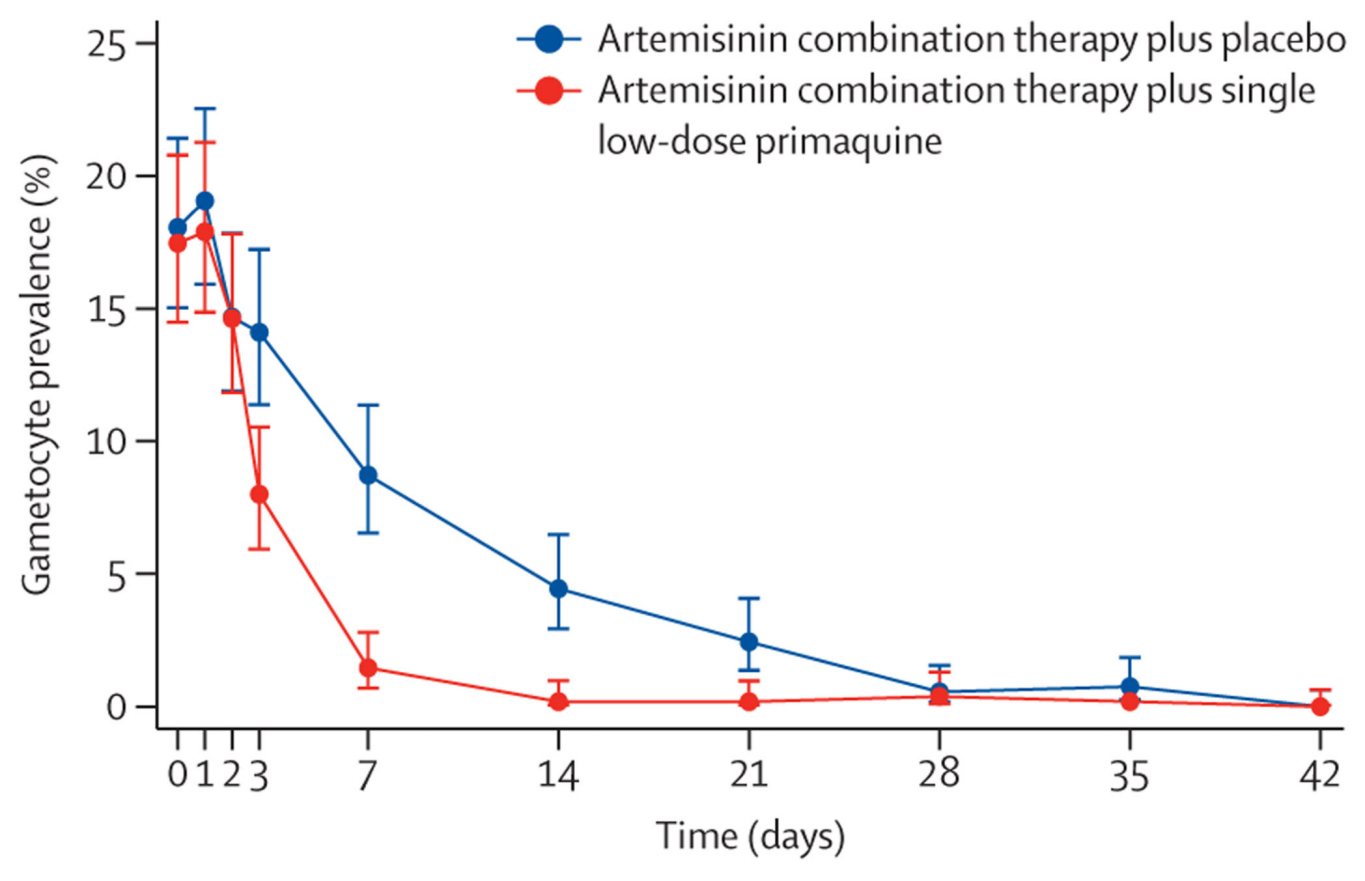
Mean gametocyte carriage over time in patients treated with artemisinin combination therapy plus either primaquine or placebo Error bars indicate 95% CI.

201 (18%) of 1136 (one patient had no data) children had day 0 gametocytes; of these, 12 children from Uganda had gametocytaemia and tested negative for asexual parasites, representing 12 (8%) of 151 children in total who tested negative for asexual parasites. After treatment, an initial increase in gametocyte carriage was followed by a decline; the gametocyte carriage was significantly lower in the artemisinin combination therapy plus single low-dose primaquine group than in the artemisinin combination therapy plus placebo group on day 3 (8% *vs* 14%, p=0·0012), day 7 (1·5% *vs* 9%, p<0·0001), day 14 (0·2% *vs* 4·4%, p<0·0001), and day 21 (0·2% *vs* 2·4%, p=0·0011; figure 3). Stratifying by artemisinin combination therapy produced similar results (appendix pp 22–23). Detailed analyses will be presented elsewhere.

## Discussion

Artemisinin combination therapy combined with single low-dose primaquine, dosed by age, was well tolerated and safe in a population of young children from Uganda and the Democratic Republic of the Congo with acute uncomplicated falciparum malaria, regardless of G6PD status. These results also reconfirm earlier findings of rapid reductions in gametocyte carriage after single low-dose primaquine compared with placebo.^9,10,12^

Single low-dose primaquine was safe in children with G6PD deficiency, who mostly had mild anaemia on admission, and many of whom had a concomitant haemoglobinopathy. Compared with the non-G6PD-deficient group, patients in the G6PD-deficient group had slightly lower mean baseline haemoglobin concentrations; however, G6PD status did not affect the initial decrease in haemoglobin concentrations in patients treated with either primaquine or placebo. Sickle cell trait, sickle cell disease, and α+-thalassaemia are associated with different degrees of anaemia, but only sickle cell trait affected the initial decrease in haemoglobin concentration, resulting in a lower fractional decrease compared with normal haemoglobin type independent of the use of single low-dose primaquine.

Although G6PD deficiency protects against falciparum malaria, especially severe malaria, a substantial proportion of children from sub-Saharan Africa with malaria will have G6PD deficiency, emphasising the importance of the favourable safety profile of single low-dose primaquine shown in this study. A− is the most common G6PD-deficiency variant in sub-Saharan Africa and is milder than the G6PD Santa Maria and G6PD Mediterranean variants, which are also present. Single low-dose primaquine was well tolerated in patients with severe southeast Asian variants of G6PD deficiency,^13,15^ and is likely to be well tolerated in the G6PD Santa Maria and G6PD Mediterranean variants.

The use of single low-dose primaquine is recommended for low-transmission areas, where its gametocytocidal effect on transmission is larger than in high-transmission settings. Although malaria transmission remains high in large parts of sub-Saharan Africa, there are currently large areas where transmission has been substantially reduced and where single low-dose primaquine is likely to have a positive effect. Perhaps more importantly, single low-dose primaquine could be an invaluable tool in the containment of artemisinin-resistant *P falciparum*, which has now emerged in Rwanda^25^ and Uganda.^26^ Studies indicate that the transmissibility of artemisinin-resistant *P falciparum* is greater than that of artemisinin-sensitive *P falciparum*,^27^ so single low-dose primaquine could be useful in reducing the spread of these resistant infections.

We chose a clinically relevant safety endpoint of profound anaemia or severe, life-threatening anaemia. These conditions are indications for a blood transfusion, a major and costly intervention that has risks of transmitting HIV, hepatitis B, and hepatitis C,^28^ and causing transfusion reactions. In our study, very few patients developed life-threatening severe anaemia (0·26% overall), and only around 1% of patients received a blood transfusion, half of whom were not G6PD deficient. In contrast to its perceived risks, primaquine is remarkably safe; only 14 deaths have been reported in patients infected with *Plasmodium vivax* who received daily primaquine, giving an estimated risk of around two deaths per one million treatments.^29^

Our study had few exclusion criteria to reflect real-life conditions. A sizeable minority of patients from Uganda had a positive rapid diagnostic test for malaria but were malaria-slide negative, probably due to the persistence of *P falciparum* HRP2, submicroscopic parasitaemia, or both, although some patients might have had a non-malarial fever. Two patients required early blood transfusion, and approximately 8% of patients were gametocytaemic and were able to transmit malaria. Including such patients is relevant because many clinics rely on rapid diagnostic tests to diagnose malaria, and non-malarial infections can cause haemolysis in patients with G6PD deficiency.^30^ Despite setting a haemoglobin concentration of less than 6 g/dL as the exclusion cutoff, the mean starting haemoglobin concentration was relatively high (10 g/dL) and moderate anaemia was correspondingly low (around 7%), which is consistent with a meta-analysis of anaemia in uncomplicated malaria infections.^5^ These findings could reflect greater community awareness of malaria, resulting in early presentation. Our study included children with sickle cell disease, in whom median baseline haemoglobin concentrations are usually 7–8 g/dL; however, too few were enrolled to draw inferences about the safety of single low-dose primaquine in this important group, who are prone to falciparum-induced haemolytic crises.

Methaemoglobinaemia and abdominal pain are known dose-related side-effects of primaquine. However, treatment with single low-dose primaquine had minimal effect on methaemoglobin concentrations, and the highest concentrations observed were far lower than the 15–20% at which cyanosis is detected clinically. No children developed severe abdominal pain, and rates of early vomiting requiring retreatment were low, with no differences between patients who received single low-dose primaquine and those who received placebo.

Single low-dose primaquine was dosed by age, using a regimen^3^ that intentionally underdosed the youngest 14 children (aged between 6 months and 11 years) with 1·25 mg as a precaution. The overall median dose was 0·21 mg/kg, which is lower than the target dose of 0·25 mg/kg recommended by WHO, and ranged from 0·07 mg/kg to 0·4 mg/kg. The dose was not an independent factor explaining the initial decrease in haemoglobin concentration, and pharmacokinetic analyses (to be published elsewhere) show that exposures are proportional to the administered dose and are lower in younger children than in older children, consistent with the findings of Goncalves and colleagues.^31^ Although our data support the use of our age-based, single low-dose primaquine regimen, the good tolerability and pharmacokinetic data suggest that higher doses, closer to the 0·25 mg/kg target dose, would also be well tolerated.

The main limitation of this study was not reaching the sample size we had originally planned. The rate of our robust and clinically important anaemia outcome was very low (approximately 0·7%) among patients with G6PD deficiency who were treated with single low-dose primaquine; with this low event rate, much larger studies would be needed to show the non-inferiority of single low-dose primaquine compared with placebo.

In conclusion, single low-dose primaquine, dosed by age and at a median dose slightly lower than that recommended by WHO, reduced gametocyte carriage and was similarly well tolerated by children who are *P falciparum*-infected with or without G6PD deficiency. Our data are reassuring for malaria control programmes in sub-Saharan Africa at a time when artemisinin-resistant *P falciparum* is emerging, and support age-based dosing as a practical option.

## Supplementary Material

Supplementary appendix

## Figures and Tables

**Table 1:** Baseline characteristics

	Artemether-lumefantrine plussingle low-dose primaquine (n=286)		Artemether-lumefantrine plus placebo (n=286)	Dihydroartemisinin-piperaquine plus single low-dose primaquine (n=283)	Dihydroartemisinin-piperaquine plus placebo (n=282)	All patients (n=1137)
Age, years (median, IQR, range)	5 (3-7, 0-5-11)		5 (3-7, 0-5-11)	5 (3-8, 0-5-11)	5 (2-8, 0-5-11)	5 (3-8, 0-5-11)
Sex						
Male	164/286 (57·3%)		172/286 (60·1%)	173/283 (61·1%)	158/282 (56·0%)	667/1137 (58·7%)
Female	122/286 (42·7%)		114/286 (39·9%)	110/283 (38·9%)	124/282 (44·0%)	470/1137 (41·3%)
Weight (kg)	17-0 (12-8-22-5)		17-0 (12-0-22-0)	17-0 (12-9-23-4)	16-5 (12-7-22-0)	17-0 (12-7-22-1)
Length of illness (days)	2(1-3)		2 (2-3)	2(2-3)	2(2-3)	2 (2-3)
Dose of primaquine or placebo, mg/kg (median, IQR, range)	0-21 (0-17-0-25, 0-11-0-37)		0-21 (0-18-0-25, 0-12-0-35)	0-20 (0-17-0-25, 0-07-0-41)	0-22 (0-18-0-25, 0-07-0-38)	0-21 (0-17-0-25, 0-07-0-41)
Temperature (*°*C)*	37-1 (36-6-38-0)		37-1 (36-6-38-0)	37-1 (36-6-38-0)	37-0 (36-5-37-8)	37-1 (36-6-38-0)
Febrile (core temp >38*°*C)*	80/286 (28·0%)		74/286 (25·9%)	75/283 (26·5%)	63/281 (22·4%)	292/1136 (25·7%)
Respiratory rate (breaths per min)*	28 (27-32)		28 (28-32)	28 (26-32)	28 (27-32)	28 (27-32)
Heart rate (beats per min)*	124 (109-137)		124 (110-136)	124 (107-135)	124 (109-136)	124 (109-136)
Jaundice	5/286 (1·7%)		10/286 (3·5%)	6/283 (2·1%)	3/282 (1·1%)	24/1137 (2·1%)
Severe pallor	2/286 (0·7%)		1/286 (0·7%)	0/283 (0%)	0/282 (0%)	3/1137 (0·3%)
MUAC (cm)	15-6 (14-5-17-0)		15-6 (14-5-17-0)	15-8 (14-5-17-0)	15-5 (14-5-16-8)	15-6 (14-5-17-0)
Nutritional statust						
Not malnourished	282/286 (98·6%)		285/286 (99·7%)	280/283 (98·9%)	282/282 (100%)	1129/1137 (99·3%)
Moderate acute malnutrition	3/286 (1·0%)		1/286 (0·3%)	3/283 (1·1%)	0/282 (0%)	7/1137 (0·6%)
Severe acute malnutrition	1/286 (0·3%)		0/286(0%)	0/283 (0%)	0/282 (0%)	1/1137 (0·1%)
Signs of wasting	4/286 (1·4%)		1/286 (0·3%)	0/283 (0%)	1/282 (0·4%)	6/1137 (0·5%)
Bipedal oedema	2/286 (0·7%)		1/286 (0·3%)	0/283 (0%)	0/282(0%)	3/1137 (0·3%)
Hepatomegaly	16/286 (5·6%)		15/286 (5·2%)	14/283 (4·9%)	11/282 (3·9%)	56/1137 (4·9%)
Splenomegaly	69/286 (24·1%)		58/286 (20·3%)	68/283 (24·0%)	71/282 (25·2%)	266/1137 (23·4%)
Haemoglobin (g/dL; mean, SD)	10-5 (1-7)		10-7 (1-5)	10-7 (1-6)	10-5 (1-6)	10-6 (1-6)
Haemoglobin <8 g/dL	20/286 (7-0%)		15/286 (5·2%)	19/283 (6·7%)	22/282 (7·8%)	76/1137 (6·7%)
Genotypic G6PD status						
G6PD-normal males	101/281 (35·9%)		117/285 (41·1%)	99/276 (35·9%)	101/278 (36·3%)	418/1120 (37·3%)
G6PD-deficient hemizygous males	60/281 (21·4%)		55/285 (19·3%)	70/276 (25·4%)	54/278 (19·4%)	239/1120 (21·3%)
G6PD-normal females	75/281 (26·7%)		69/285 (24·2%)	75/276 (27·2%)	80/278 (28·8%)	299/1120 (26·7%)
G6PD·deficient homozygous females	13/281 (4·6%)		12/285 (4·2%)	8/276 (2·9%)	12/278 (4·3%)	45/1120 (4·0%)
Heterozygous females	32/281 (11·4%)		32/285 (11·2%)	24/276 (8·7%)	31/278 (11·2%)	119/1120 (10·6%)
α-Thalassaemia status						
Not thalassaemic		132/282 (46·8%)	139/284 (48^9%)	124/272 (45·6%)	136/278 (48·9%)	531/1116 (47·6%)
Single a deletion		124/282 (44·0%)	122/284 (43·0%)	122/272 (44·9%)	109/278 (39·2%)	477/1116 (42^7%)
Double a deletion		26/282 (9·2%)	23/284 (8·1%)	26/272 (9·6%)	33/278 (11·9%)	108/1116 (9·7%)
Sickle cell status						
No sickle cell trait or disease		248/282 (87·9%)	243/285 (85·3%)	234/278 (84·2%)	231/277 (83·4%)	956/1122 (85·2%)
Sickle cell trait		33/282 (11·7%)	42/285 (14·7%)	42/278 (15·1%)	46/277 (16·6%)	163/1122 (14·5%)
Sickle cell disease		1/282 (0·4%)	0/285 (0%)	2/278 (0·7%)	0/277 (0%)	3/1122 (0·3%)
Asexual parasite result in first 24 h						
Negative		37/286 (12·9%)	35/286 (12·2%)	44/283 (15·5%)	35/281 (12·5%)	1514/1136 (13·3%)
KIMORU		0/135 (0%)	0/137 (0%)	0/134 (0%)	0/132 (0%)	0/538 (0%)
MRRH		37/151 (24·5%)	35/149 (23·5%)	44/149 (29·5%)	35/149 (23·5%)	151/598 (25·3%)
Positive *(P falciparum, P malariae,* and *P ovale)*		249/286 (87·1%)	251/286 (87·8%)	239/283 (84·5%)	246/281 (87·2%)	985/1136 (86·6%)
*Plasmodium falciparum* asexual parasitaemia, number per μL (geometric mean, SD, range)		13 873 (15, 7-1 397 958)	12 345 (15, 14-1 100 764	14 148 (16, 12-668 770)	18 848 (13, 7-2 172 060)	14 599 (15, 7-2 172 060)
Gametocyte carriage						
Negative		231/286 (80·8%)	235/286 (82·2%)	238/283 (84·1%)	228/281 (81·1%)	932/1136 (82·0%)
Positive (*P falciparum, P malariae,* and *P ovale*)		55/286 (19·2%)	51/286 (17·8%)	45/283 (15·9%)	53/281 (18·9%)	204/1136 (18·0%)
*P falciparum* gametocyte carriage		55/286 (19·2%)	50/286 (17·5%)	44/283 (15·5%)	52/281 (18·5%)	201/1136 (17·7%)
*P falciparum* gametocytaemia, number per μL (geometric mean, SD, range)		53 (4, 8-1778)	37 (3, 8-2028)	50(4,10-2317)	44 (3, 9-2888)	45 (4, 8-2888)

Data are median (IQR), n (%), or n/N (%), unless otherwise stated. MUAC=middle-upper-arm circumference. G6PD=glucose-6-phosphate dehydrogenase. KIMORU=Kinshasa Mahidol Oxford Research Unit. MRRH=Mbale Regional Referral Hospital.

* One patient did not have vital sign data.

† Not malnourished is defined as MUAC ≥12·5 cm; moderate acute malnutrition is defined as a MUAC of 11·5 cm to less than 12·5 cm; severe acute malnourishment is defined as MUAC <11·5 cm.

‡ All negative slides are from MRRH. 16 patients were G6PD-deficient, 20 were heterozygous girls, 114 were G6PD-normal, and one patient had unknown G6PD status.

**Table 2:** Summary of clinically reported and laboratory adverse events

	Artemether- lumefantrine plus single low-dose primaquine	Artemether- lumefantrine plus placebo	Dihydroartemisinin- piperaquine plus single low-dose primaquine	Dihydroartemisinin- piperaquine plus placebo	All patients	p value*
Early vomiting						
Day 0	7/286 (2·4%)	11/286 (3·8%)	11/283 (3·9%)	11/282 (3·9%)	40/1137 (3·5%)	0·53
Day 1	5/286 (1·7%)	1/286 (0·3%)	3/283 (1·1%)	4/282 (1·4%)	13/1137 (1·1%)	··
Day 2	2/286 (0·7%)	4/286 (1·4%)	1/283 (0·4%)	1/282 (0·4%)	8/1137 (0·7%)	··
Abdominal pain						
Grade 1 or 2	7/280 (2·5%)	11/281 (3·9%)	5/280 (1·8%)	9/276 (3·3%)	32/1117 (2·9%)	0·16
Nausea						
Grade 1 or 2	6 (2·1%)	1 (0·3%)	1 (0·4%)	2 (0·7%)	10 (0·9%)	0·34
Diarrhoea						
Grade 1 or 2	7 (2·4%)	6 (2·1%)	7 (2·5%)	4 (1·4%)	24 (2·1%)	0·54
Headache						
Grade 1 or 2	15/280 (5·4%)	13/279 (4·7%)	14/279 (5·0%)	13/272 (4·8%)	55/1110 (5·0%)	0·78
Grade 3 or 4	0/280 (0%)	0/279 (0%)	0/279 (0%)	1/272 (0·4%)	1/1110 (0·1%)	0·50
Neutropenia (<400 neutrophils per μL)	2 (0·7%)	2 (0·7%)	0 (0%)	0 (0%)	4 (0·4%)	1·0
Thrombocytopenia (<50 000 platelets per μL)	4 (1·4%)	3 (1·0%)	1 (0·4%)	3 (1·1%)	11 (1·0%)	0·77
AST≥3 x ULN†	3/213 (1·4%)	2/217 (0·9%)	3/219 (1·4%)	1/210 (0·5%)	9/859 (1·1%)	0·51
ALT≥3 x ULN†	0/213 (0%)	2/217 (0·9%)	2/219 (0·9%)	0/210 (0%)	4/859 (0·5%)	1·0
Total bilirubin ≥2 x ULN†	0/212 (0%)	1/216 (0·5%)	1/216 (0·5%)	0/208 (0%)	2/852 (0·2%)	1·0
Treatment·emergent acute kidney injury‡						
Grade 1	23/186 (12·4%)	17/187 (9·1%)	31/183 (16·9%)	30/185 (16·2%)	101/741 (13·6%)	0·46
Grade 2	15/186 (8·1%)	16/187 (8·6%)	16/183 (8·7%)	15/185 (8·1%)	62/741 (8·4%)	1·0
Grade 3	12/186 (6·5%)	22/187 (11·8%)	14/183 (7·7%)	10/185 (5·4%)	58/741 (7·8%)	0·5

Data are n (%) or n/N (%). ALT=alanine aminotransferase. AST=aspartate transaminase. ULN=upper limit of normal. *Comparisons are between artemisinin combination therapy plus single low-dose primaquine and artemisinin combination therapy plus placebo. †ULNs are AST 60 in males; 50 in females; ALT 55 in males; 45 in females; and total bilirubin 1·0 mg/dL. ‡Grade 1: creatinine concentration 1·5–1·9 × day 0 concentration. Grade 2: creatinine concentration 2·0–2·9 × day 0 concentration. Grade 3: creatinine concentration 3 or more × day 0 concentration. Denominators vary because the routine biochemistry was done in a subset of patients.

## Research in context

### Evidence before this study

A single low dose of primaquine (0·25 mg/kg) was recommended by WHO in 2012 as gametocytocidal therapy in falciparum malaria at a time when the evidence base for its use was small. We searched PubMed from database inception to July 27, 2022, with no language restrictions, for studies assessing the transmission-blocking effect and tolerability of primaquine using the search terms ([Primaquine] AND [G6PD deficiency] OR [Haemolysis]) and ([Primaquine] AND [Gametocytocidal] OR [Gametocytes] OR [Transmission]). Since 2012, several studies on the efficacy of single low-dose primaquine have shown a dose-dependent effect on the reduction of gametocyte carriage and mosquito infectivity. Clinical studies support the safety of single low-dose primaquine in patients with glucose-6-phosphate dehydrogenase (G6PD) deficiency who are infected with *Plasmodium falciparum*, but these studies generally included mostly older children (ie, 5 years old and older) and adults with relatively high baseline haemoglobin concentrations. One key issue remains the safety of the routine use of single low-dose primaquine in young children from Africa with G6PD deficiency and acute *P falciparum* infection.

### Added value of this study

This large, multicentre, placebo-controlled study, which recruited only children, included many patients with a genotypically confirmed G6PD deficiency. The inclusion and exclusion criteria were less restrictive than those of other studies to try to reflect real-life field conditions. Of note is that the haemoglobin exclusion cutoff was less than 6 g/dL, in contrast to 8 g/dL or more in other studies. Moreover, some studies excluded patients with G6PD deficiency and others included only asymptomatic individuals. Our primary endpoint is highly relevant to clinical practice and is a clearer endpoint than the change in haemoglobin concentration over time that is reported elsewhere. Only three patients met our primary endpoint, and single low-dose primaquine had similar tolerability to placebo.

### Implications of all the available evidence

Our study adds substantial evidence regarding the safety of single low-dose primaquine in children with G6PD deficiency and acute *P falciparum* infection who were treated with two commonly used antimalarial regimens. These results support the implementation of routine, single low-dose primaquine as gametocytocidal treatment in children from sub-Saharan Africa in the contexts of falciparum-malaria elimination and the containment of recently emerged, artemisinin-resistant *P falciparum* in the region.

## Data sharing

Deidentified individual participant data and relevant supplementary data and documents (eg, data dictionary, protocol, and participant information sheet) will be available immediately after publication of the key papers (main and pharmacokinetic papers) to applicants who provide a sound proposal to the Mahidol Oxford Tropical Medicine Research Unit Data Access Committee (datasharing@tropmedres.ac). A data access agreement will be put in place before sharing.
