# Supplementary material for: Safety of age-dosed, single low-dose primaquine in children with glucose-6-phosphate dehydrogenase deficiency who are infected with Plasmodium falciparum in Uganda and the Democratic Republic of the Congo: a randomised, double-blind, placebo-controlled, non-inferiority trial
Source: Lancet Infect Dis. Author manuscript; Available in PMC 2025 Oct 12. (PMC7618244; doi:10.1016/S1473-3099(22)00658-2)

THE LANCET
Infectious Diseases

Supplementary appendix

This appendix formed part of the original submission and has been peer reviewed. We post it as supplied by the authors.

Supplement to: Taylor WR, Olupot-Olupot P, Onyamboko MA, et al. Safety of age-dosed, single low-dose primaquine in children with glucose-6-phosphate dehydrogenase deficiency who are infected with Plasmodium falciparum in Uganda and the Democratic Republic of the Congo: a randomised, double-blind, placebo-controlled, non-inferiority trial. *Lancet Infect Dis* 2022; published online Nov 30. https://doi.org/10.1016/S1473-3099(22)00658-2.

Table of contents.

Table of contents. 1
Study schedule. 2
Randomisation scheme 3
Photographs of the dosing cards and envelope 4
Trial profiles by site. 7
Baseline characteristics of participants in Kinshasa, DR Congo 9
Baseline characteristics of participants in Mbale, Uganda 11
Analysis of primary end point 13
The absolute and fractional falls in haemoglobin from baseline to the nadir haemoglobin ≤ 14 days of follow up. 14
Factors examined for an independent association with haemoglobin fractional fall from baseline to nadir ≤ 14 days 15
Mean reticulocyte counts over time for all patients. 16
Changes in methaemoglobin over time for all patients combined in the single low dose primaquine and placebo arms. 17
Changes in methaemoglobin concentration over time by G6PD status and primaquine allocation. 18
AST concentrations overtime in all patients. 20
ALT concentrations overtime in all patients. 20
Total bilirubin concentrations overtime in all patients. 21
Creatinine concentrations overtime in all patients. 21
Details of patients who were transfused. None had sickle cell trait or disease. 22
Gametocyte carriage in children treated with artemether lumefantrine. 23
Gametocyte carriage in children treated with dihydroartemisinin piperaquine. 23

Study schedule.

Initial patient management																									**Blood volumes**	
																										**total**
Verbal consent (routine in DRC)	X																									
Brief history & examination	X																									
Hb (HemoCue)	X																								0.02	0.02
Malaria RDT	X																								0.02	0.02
Malaria blood film (Kinshasa only if malaria RDT positive)	X																								0.05	0.05
G6PD RDT qualitative	X																								0.02	0.02
	**D0**								**D1**		**D2**		**D3**		**D4**	**D5**	**D6**	**D7**	**D14**	**D21**	**D28**	**D35**	**D42**	**Drec**		
	**H0**	**H1**	**H1.5**	**H2**	**H4**	**H6**	**H8**	**H12**	**H24**	**H36**	**H48**	**H60**	**H72**	**H84**	**H96**											
Enrolled patients																										
Written consent																										
Assign study number	X																									
Open question about well being	X								X		X		X		X	X	X	X	X	X	X	X	X	X		
Symptoms checklist	X												X					X	X	X	X	X	X	X		
Weight	X																									
Vital signs	X							X	X	X	X	X	X	X	X	X	X	X	X	X	X	X	X	X		
General examination†	X																									
MetHb oximeter	X					X		X	X	X	X	X	X	X	X	X	X	X	X	X	X	X	X			
Drug administration																										
AL	X								X		X															
DHAPP	X								X		X															
Primaquine/placebo	X																									
Laboratory tests - finger sticks																										
Malaria blood films	X					X		X	X	X	X	X	X	X	X	X	X	X	X	X	X	X	X	X	0.05	1
Hb (HemoCue)						X		X	X	X	X	X	X	X	X	X	X	X	X	X	X	X	X	X	0.05	1
Haematocrit - microcentrifuge	X					X		X	X	X	X	X	X	X	X	X	X	X	X	X	X	X	X	X	0.05	1
Thin film for retic count	X												X					X	X	X	X	X	X		0.05	0.45
G6PD Biosensor	X																								0.03	0.03
Hb colour card	X												X					X			X				0.03	0.12
Filter paper samples																										
Filter paper blood spot - gametocyte qPCR	X												X					X	X	X	X	X	X		0.05	0.04
Filter paper blood spot - malaria genotype	X																	X	X	X	X	X	X	X	0.05	0.04
Filter paper molecular markers of resistance	X																								0.05	0.05
Venous sample																										
FBC EDTA#	X												X					X			X				2	8
Biochemistry-heparin‡	X												X					X			X				2	8
Lumefantrine/piperaquine (EDTA)	X												X					X			X				2	8
Primaquine*	X	X	X	X	X	X	X	X	X																0.75	6
																										**33.7**

D0 is the first day of dosing for all patients

Do investigations on D4-6 only if patient is still admitted for medical reasons

† Vital signs includes colour of lips & conjunctivae.

Do physical examination as clinically indicated post D0

Drec = day of recurrence

* Primaquine - in a subset

EDTA: G6PD activity, PCR studies for G6PD, haemoglobinopathies & CYP2D6

‡ AST, ALT, LDH, total bilirubin, conjugated bilirubin, creatinine, (store for haptoglobin + folate)

Randomisation scheme

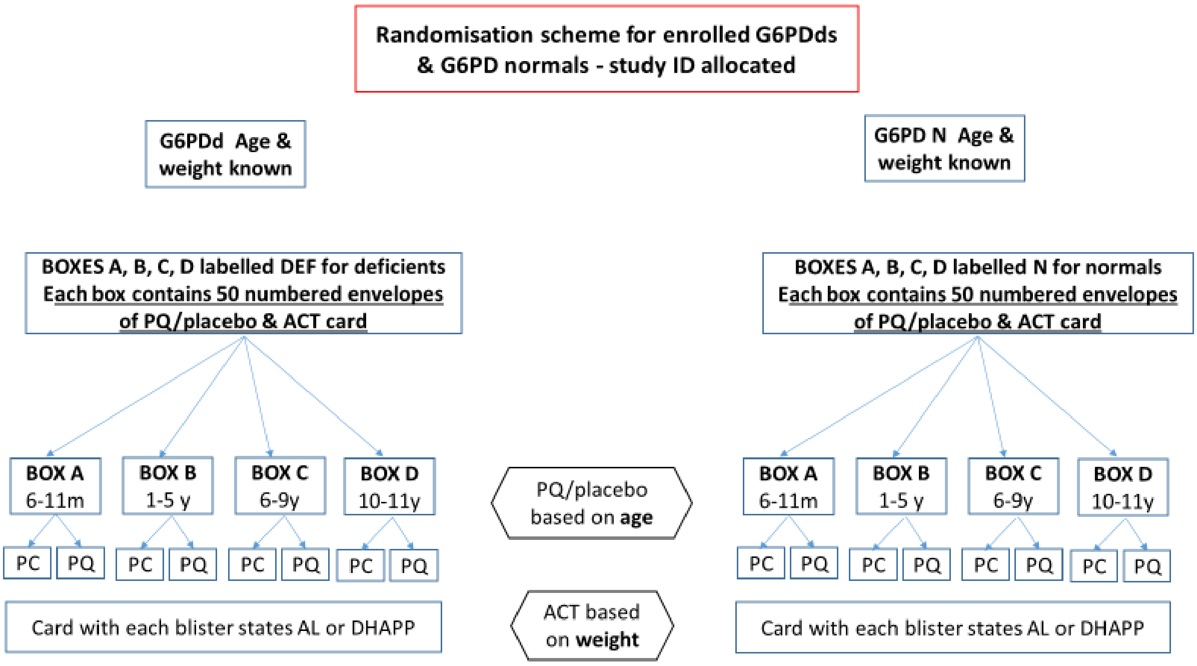

Photographs of the dosing cards and envelope.

Dosing card containing tablet strengths of either 2.5, 5 or 7.5 mg of primaquine/placebo. The reverse side shows the allocation of the artemisinin-based combination treatment. Each card was placed in a labelled sealed envelope. Illustration is for a G6PD normal patient aged 6 – 11 months (A) with the randomisation number (i.e. blinded code) 0062.

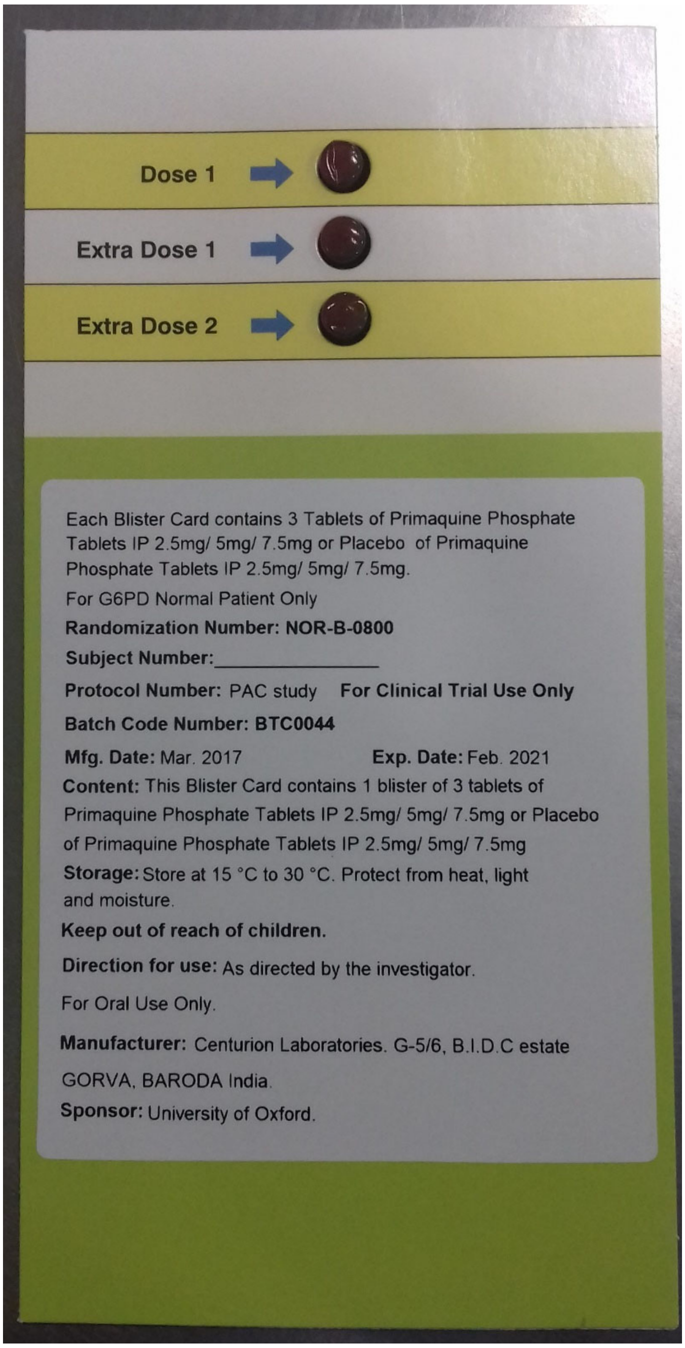

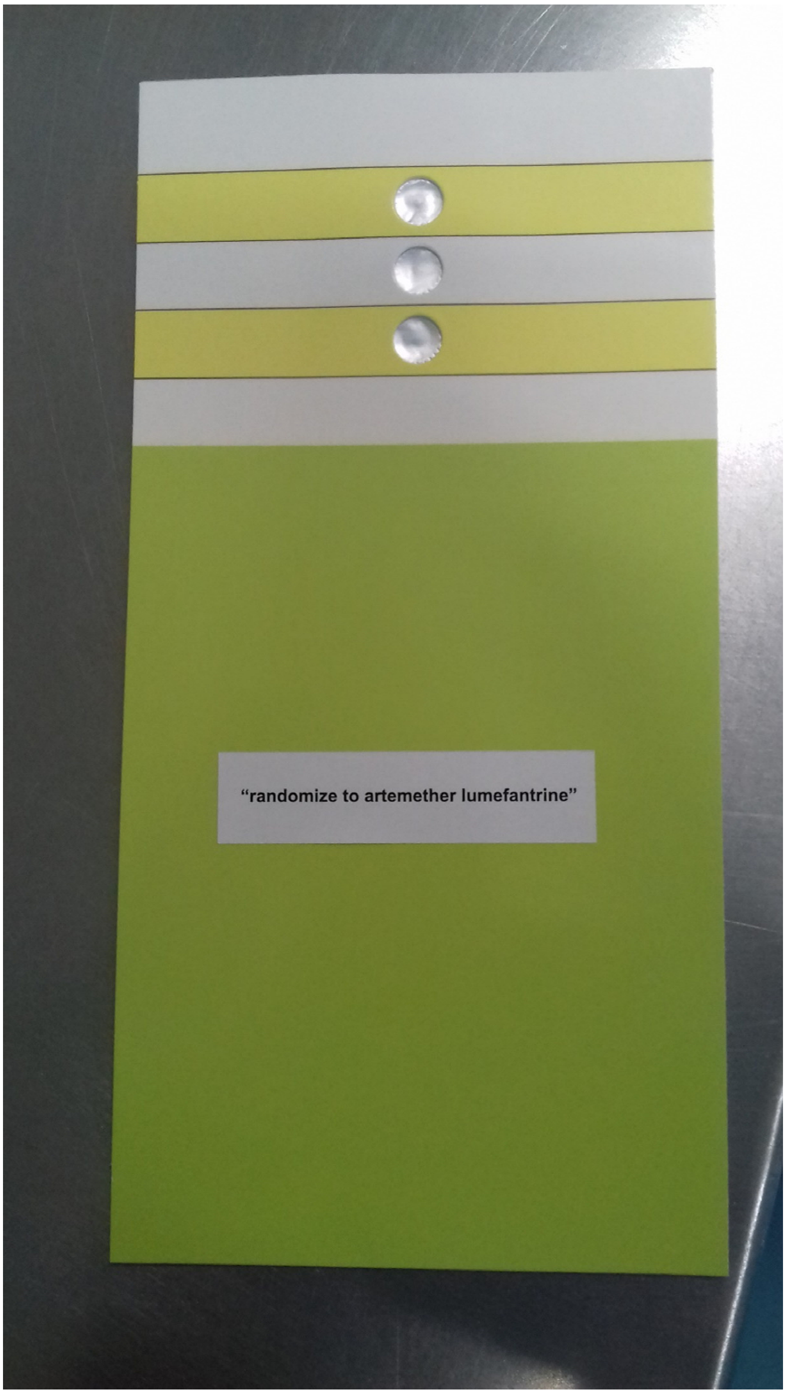
"randomize to artemether lumefantrine"

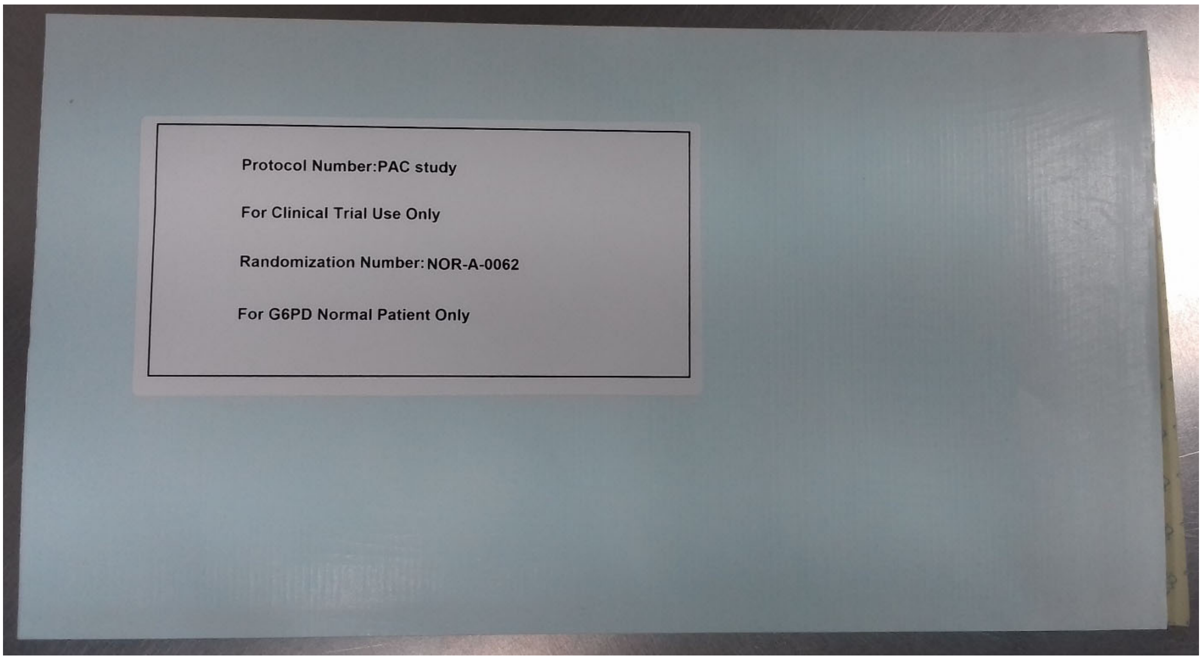
Protocol Number:PAC study
For Clinical Trial Use Only
Randomization Number: NOR-A-0062
For G6PD Normal Patient Only

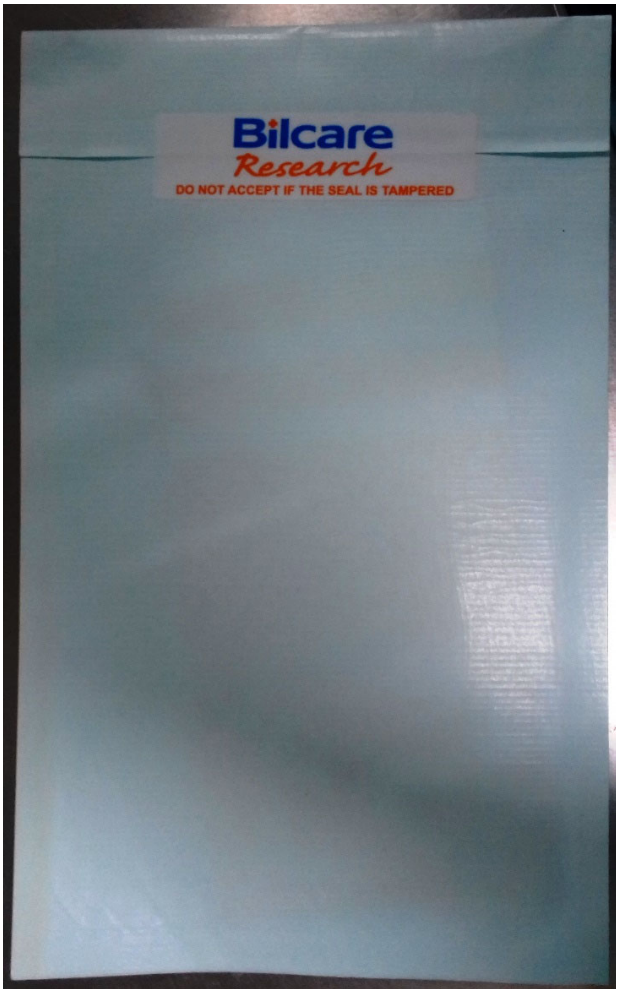
Bilcare
Research
DO NOT ACCEPT IF THE SEAL IS TAMPERED

Trial profiles by site.

KIMORU, Kinshasa.

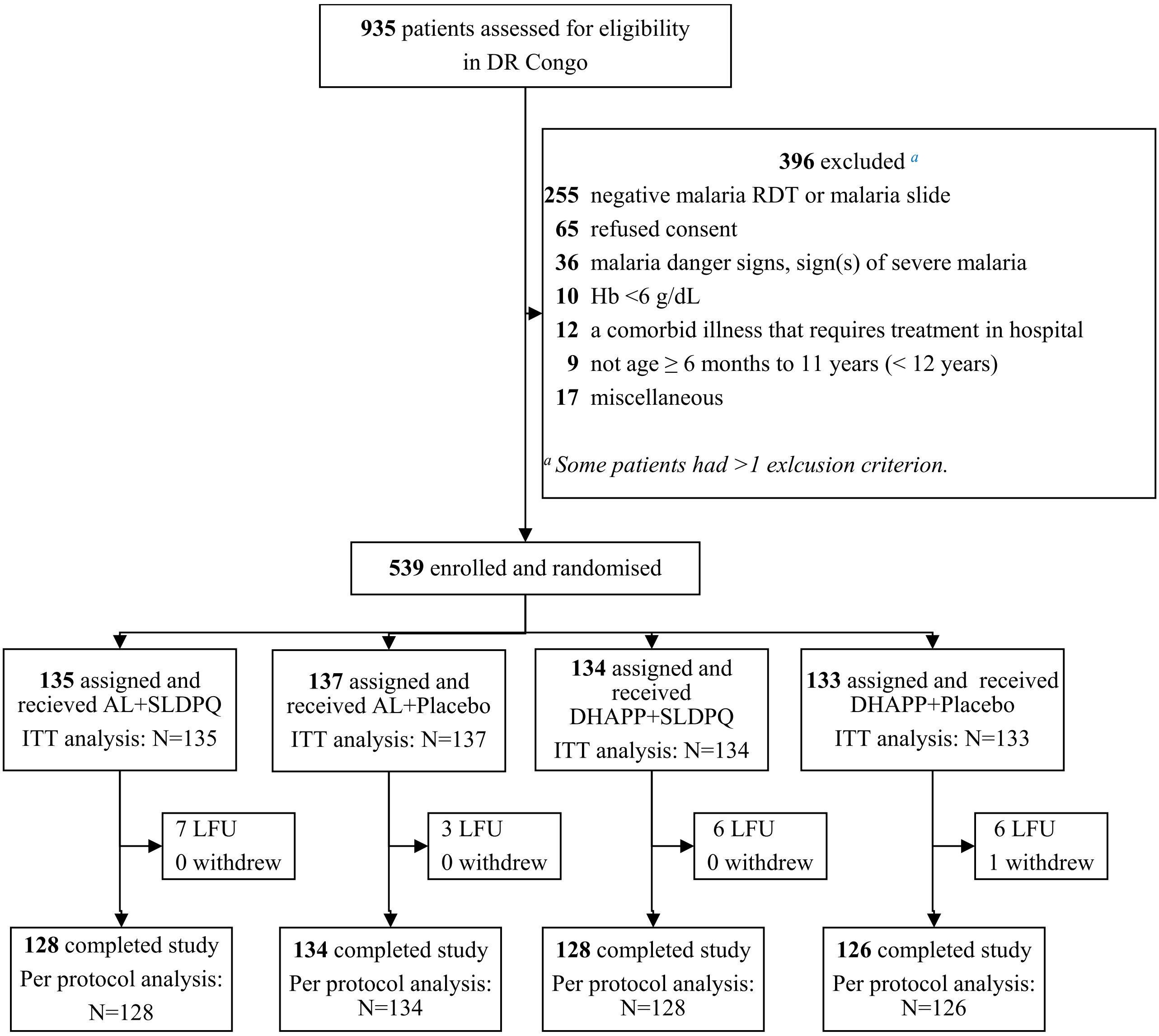

Mbale, Uganda.

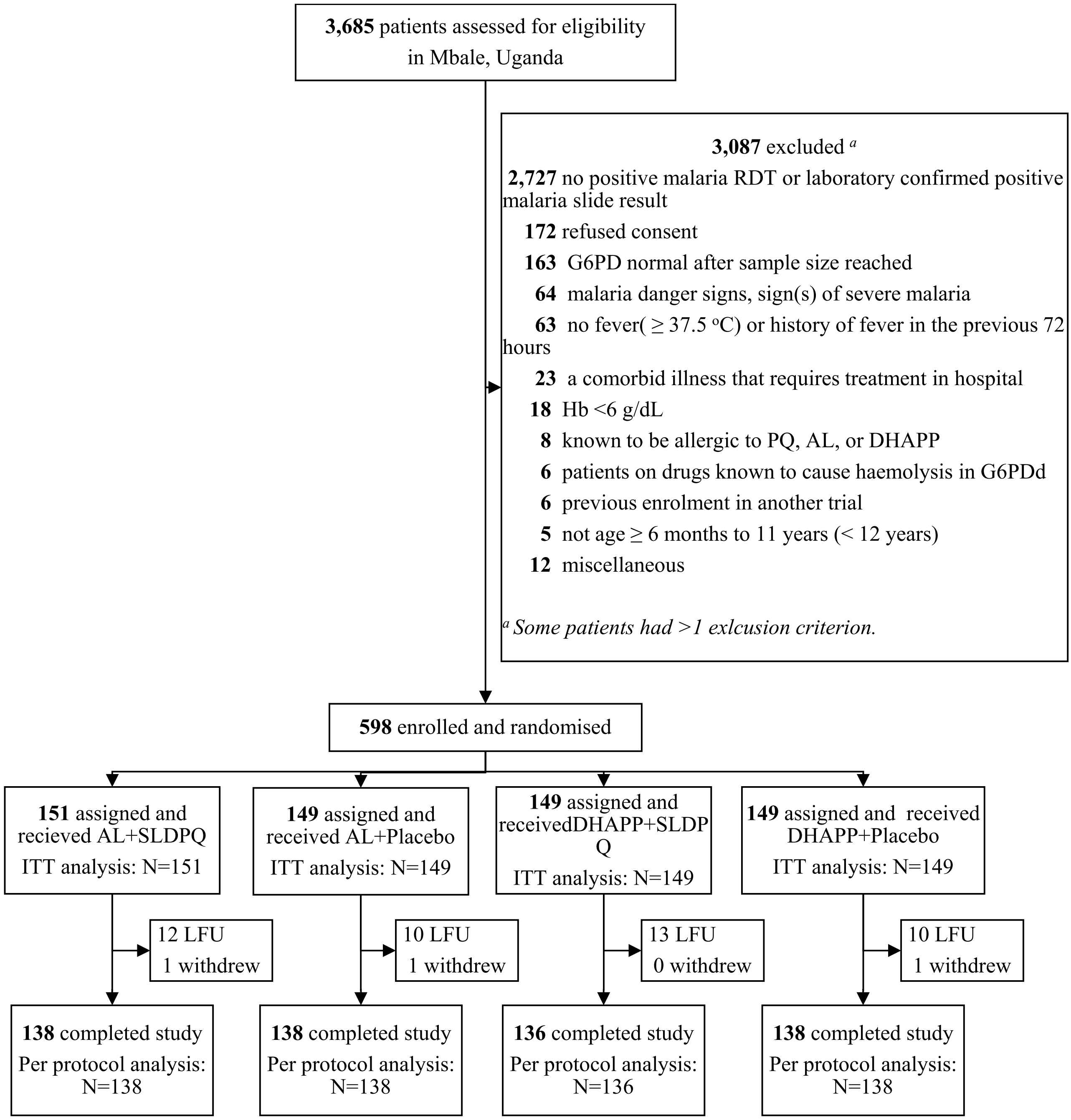

Baseline characteristics of participants in Kinshasa, DR Congo

	AL+SLDPQ (N=135)	AL+Placebo (N=137)	DHAPP+SLDPQ (N=134)	DHAPP+Placebo (N=133)	All patients (N=539)
Age (years), median (IQR; range)	6 (4, 9; 0.6 – 11)	6 (4, 8; 0.7 – 11)	7 (4, 9; 0.7 – 11)	6 (3, 9; 0.6 – 11)	6 (4, 9; 0.6 – 11)
Male, n (%)	90 (67)	92 (67)	89 (66)	89 (67)	360 (67)
Weight (kg), median (IQR)	19.0 (15.0, 25.0)	19.0 (15.0, 24.5)	19.0 (15.0, 25.5)	19.0 (14.0, 24.5)	19.0 (14.5, 25.0)
Length of illness before treatment (days), median (IQR)	2 (1, 2)	2 (1, 2)	2 (1, 2)	2 (1, 2)	2 (1, 2)
Mg/kg primaquine (PQ)/PQ placebo, median (IQR; range)	0.21 (0.17, 0.25; 0.11 – 0.36)	0.22 (0.18, 0.25; 0.13 – 0.35)	0.21 (0.17, 0.25; 0.07 – 0.33)	0.22 (0.18, 0.26; 0.13 – 0.38)	0.22 (0.18, 0.25; 0.07 – 0.38)
Temperature (*ºC*) *, median (IQR)	36.8 (36.4, 37.5)	36.8 (36.5, 37.6)	36.8 (36.4, 37.5)	36.8 (36.4, 37.4)	36.8 (36.4, 37.5)
Febrile (core temp ≥ 38*ºC*) *, n/N (%)	19/135 (14)	22/137 (16)	24/134 (18)	17/132 (13)	82/538 (15)
Respiratory rate (*bpm*) *, median (IQR)	28 (28, 32)	28 (28, 32)	28 (28, 32)	28 (28, 32)	28 (28, 32)
Heart rate (*bpm*) *, median (IQR)	117 (102, 135)	120 (104, 133)	117 (102, 133)	120 (106, 133)	119 (103, 133)
Jaundice, n (%)	1 (0.7)	0 (0)	0 (0)	0 (0)	1 (0.2)
Severe pallor, n (%)	1 (0.7)	0 (0)	0 (0)	0 (0)	1 (0.2)
MUAC (cm)	16.0 (15.0, 17.0)	16.0 (14.8, 17.5)	16.1 (15.0, 17.3)	15.6 (15.0, 17.2)	16.0 (15.0, 17.3)
Nutritional status[a], n (%)					
Not malnourished	132 (98)	136 (99)	132 (99)	133 (100)	533 (99)
Moderate acute malnutrition (MAM)	2 (1)	1 (0.7)	2 (1)	0 (0)	5 (1)
Severe acute malnutrition (SAM)	1 (0.7)	0 (0)	0 (0)	0 (0)	1 (0.2)
Signs of wasting, n (%)	2 (1.5)	0 (0)	0 (0)	0 (0)	2 (0.4)
Bipedal oedema, n (%)	1 (0.7)	0 (0)	0 (0)	0 (0)	1 (0.2)
Hepatomegaly, n (%)	11 (8)	8 (6)	8 (6)	9 (7)	36 (7)
Splenomegaly, n (%)	63 (47)	47 (34)	63 (47)	65 (49)	238 (44)
Haemoglobin (g/dL), mean (SD)	10.3 (1.5)	10.5 (1.4)	10.6 (1.6)	10.3 (1.5)	10.4 (1.5)
Haemoglobin < 8 g/dL, n (%)	10 (7)	8 (6)	10 (7.5)	10 (7.5)	38 (7.1)
Genotypic G6PD status, n (%)	(N=134)	(N=137)	(N=134)	(N=132)	(N=537)
G6PD normal males	43 (32)	47 (34)	39 (29)	44 (33)	173 (32)
G6PDd hemizygous males	47 (35)	45 (33)	50 (37)	44 (33)	186 (35)
G6PD normal females	20 (15)	22 (16)	29 (22)	21 (16)	92 (17)
G6PDd homozygous females	12 (9)	9 (7)	7 (5)	10 (8)	38 (7)
G6PDd heterozygous females	12 (9)	14 (10)	9 (7)	13 (10)	48 (9)
α-thalassaemia status, n (%)	(N=135)	(N=136)	(N=130)	(N=132)	(N=533)

Not thalassaemic	55 (41)	55 (40)	47 (36)	51 (39)	208 (39)
Silent carrier (-α/αα)	64 (47)	64 (47)	66 (51)	61 (46)	255 (48)
Thalassaemia trait (-α/-α)	16 (12)	17 (13)	17 (13)	20 (15)	70 (13)
Sickle cell status, n (%)	(N=135)	(N=137)	(N=134)	(N=132)	(N=538)
No sickle cell (HbAA)	112 (83)	105 (77)	113 (84)	105 (80)	435 (81)
Sickle cell trait (HbAS)	23 (17)	32 (23)	20 (15)	27 (20)	102 (19)
Sickle cell disease (HbSS)	0 (0)	0 (0)	1 (1)	0 (0)	1 (0.2)
Asexual parasite result within 24 hours, n/N (%)					
Negative	0/135 (0)	0/137 (0)	0/134 (0)	0/132 (0)	0/538 (0)
Positive (Pf/Pm/Po)	135/135 (100)	137/137 (100)	134/134 (100)	132/132 (100)	538/538 (100)
Pf asexual positivity within 24 hours, n/N (%)	135/135 (100)	137/137 (100)	134/134 (100)	132/132 (100)	538/538 (100)
Pf asexual parasitaemia (/µL) within 24 hours, geometric mean (range)	(N=135) 17,214 (11; 32 – 892,514)	(N=137) 14,075 (11; 14 – 976,100)	(N=134) 17,539 (11; 34 – 652,806)	(N=132) 23,958 (8; 28 – 578,765)	(N=538) 17818 (10; 14 – 976,100)
Sexual gametocyte result, n/N (%)					
Negative	95/135 (70)	95/137 (69)	102/134 (76)	96/132 (73)	388/538 (72)
Positive (Pf/Pm/Po)	40/135 (30)	42/137 (31)	32/134 (24)	36/132 (27)	150/538 (28)
Pf gametocyte carriage, n/N (%)	40/135 (30)	41/137 (30)	31/134 (23)	36/132 (27)	148/538 (28)
Pf gametocytaemia (/µL), geometric mean (range)	(N=40) 44 (4; 8 – 1,700)	(N=41) 27 (2; 8 – 114)	(N=31) 32 (3; 10 – 321)	(N=36) 37 (3; 9 – 689)	(N=148) 35 (3; 8 – 1,700)

[*] *There was 1 patient who did not have vital sign data.*
[a] *Malnutrition status definition: not malnourished (MUAC ≥ 12.5 cm), MAM (MUAC 11.5 – < 12.5 cm) and SAM (MUAC < 11.5 cm)*

Baseline characteristics of participants in Mbale, Uganda

	AL+SLDPQ (N=151)	AL+Placebo (N=149)	DHAPP+SLDPQ (N=149)	DHAPP+Placebo (N=149)	All patients (N=598)
Age (years), median (IQR; range)	4 (2, 7; 0.5 – 11)	3 (2, 6; 0.5 – 11)	4 (2, 6; 0.5 – 10)	4 (2, 7; 0.5 – 11)	4.7 (3.0; 0.5 – 11)
Male, n (%)	74 (49)	80 (54)	84 (56)	69 (46)	307 (51)
Weight (kg), median (IQR)	15.2 (10.9, 20.4)	14.5 (10.5, 19.0)	14.9 (11.4, 19.4)	15.1 (11.0, 19.5)	15.0 (10.9, 19.7)
Length of illness before treatment (days), median (IQR)	3 (2, 3)	3 (2, 3)	3 (2, 3)	3 (2, 3)	3 (2, 3)
Mg/kg primaquine (PQ)/PQ placebo, median (IQR; range)	0.21 (0.17, 0.25; 0.11 – 0.37)	0.21 (0.17, 0.26; 0.12 – 0.35)	0.20 (0.17, 0.25, 0.10 – 0.41)	0.21 (0.17, 0.25; 0.07 – 0.38)	0.21 (0.17, 0.25; 0.07 – 0.41)
Temperature (*ºC*)' median (IQR)	37.5 (36.8, 38.6)	37.4 (36.7, 38.5)	37.5 (36.9, 38.4)	37.2 (36.6, 38.2)	37.4 (36.8, 38.5)
Febrile (core temp ≥ 38*ºC*) , n/N (%)	61 (40)	52 (35)	51 (34)	46 (31)	210 (35)
Respiratory rate (*bpm*) , median (IQR)	30 (26, 34)	30 (27, 32)	29 (26, 32)	29 (26, 32)	29 (26, 33)
Heart rate (*bpm*) , median (IQR)	128 (115, 138)	129 (114, 138)	127 (114, 138)	128 (112, 138)	128 (114, 138)
Jaundice, n (%)	4 (3)	10 (7)	6 (4)	3 (2)	23 (3.8)
Severe pallor, n (%)	1 (0.7)	1 (0.7)	0 (0)	0 (0)	2 (0.3)
MUAC (cm)	15.2 (14.5, 16.5)	15.4 (14.3, 16.5)	15.3 (14.2, 16.5)	15.5 (14.1, 16.5)	15.4 (14.3, 16.5)
Nutritional status[a], n (%)					
Not malnourished	150 (99)	149 (100)	148 (99)	149 (100)	596 (100)
Moderate acute malnutrition (MAM)	1 (1)	0 (0)	1 (1)	0 (0)	2 (0.3)
Severe acute malnutrition (SAM)	0 (0)	0 (0)	0 (0)	0 (0)	0 (0)
Signs of wasting, n (%)	2 (1)	1 (0.7)	0 (0)	1 (0.7)	4 (0.7)
Bipedal oedema, n (%)	1 (0.7)	1 (0.7)	0 (0)	0 (0)	2 (0.3)
Hepatomegaly, n (%)	5 (3)	7 (5)	6 (4)	2 (1)	20 (3)
Splenomegaly, n (%)	6 (4)	11 (7)	5 (3)	6 (4)	28 (5)
Haemoglobin (g/dL), mean (SD)	10.8 (1.8)	10.8 (1.6)	10.8 (1.6)	10.7 (1.8)	10.8 (1.7)
Haemoglobin < 8 g/dL, n (%)	10 (7)	7 (5)	9 (6)	12 (8)	38 (6)
Genotypic G6PD status, n (%)	(N=147)	(N=148)	(N=142)	(N=146)	(N=583)
G6PD normal males	58 (39)	70 (47)	60 (42)	57 (39)	245 (42)
G6PDd hemizygous males	13 (9)	10 (7)	20 (14)	10 (7)	53 (9)
G6PD normal females	55 (37)	47 (32)	46 (32)	59 (40)	207 (36)
G6PDd homozygous females	1 (1)	3 (2)	1 (1)	2 (1)	7 (1)
G6PDd heterozygous females	20 (14)	18 (12)	15 (11)	18 (12)	71 (12)

α-thalassaemia status, n (%)	(N=147)	(N=148)	(N=142)	(N=146)	(N=583)
Not thalassaemic	77 (52)	84 (57)	77 (54)	85 (58)	323 (55)
Silent carrier (-α/αα)	60 (41)	58 (39)	56 (39)	48 (33)	222 (38)
Thalassaemia trait (-α/-α)	10 (7)	6 (4)	9 (6)	13 (9)	38 (7)
Sickle cell status, n (%)	(N=147)	(N=148)	(N=144)	(N=145)	(N=584)
No sickle cell (HbAA)	136 (93)	138 (93)	121 (84)	126 (87)	521 (89)
Sickle cell trait (HbAS)	10 (7)	10 (7)	22 (15)	19 (13)	61 (10)
Sickle cell disease (HbSS)	1 (1)	0 (0)	1 (1)	0 (0)	2 (0)
Asexual parasite result within 24 hours, n (%)					
Negative	37 (24)	35 (23)	44 (30)	35 (23)	151 (25)
Positive (*Pf/Pm/Po*)	114 (76)	114 (77)	105 (70)	114 (77)	447 (75)
Pf asexual positivity within 24 hours, n (%)	114 (75)	114 (77)	105 (70)	113 (76)	446 (75)
Pf asexual parasitaemia (/µL) within 24 hours, geometric mean (range)	(N=114) 10,744 (21; 7 – 1,397,958)	(N=114) 10,545 (20; 25 – 1,100,764)	(N=105) 10,755 (24; 12 – 668,770)	(N=113) 14,205 (21; 7 – 2,172,060)	(N=446) 11474 (21; 7 – 2,172,060)
Sexual gametocyte result, n (%)					
Negative	136 (90)	140 (94)	136 (91)	132 (89)	545 (91)
Positive (*Pf/Pm/Po*)	15 (10)	9 (6)	13 (9)	17 (11)	53 (9)
Pf gametocyte carriage, n (%)	15 (10)	9 (6)	13 (9)	16 (11)	82 (14)
Pf gametocytaemia (/µL), geometric mean (range)	(N=15) 87 (5; 9 – 1,778)	(N=9) 143 (7; 16 – 2,028)	(N=13) 145 (6; 17 – 2,317)	(N=16) 64 (5; 9 – 2,888)	(N=53) 98 (5; 9 – 2,888)

[a] Malnutrition status definition: not malnourished (MUAC ≥ 12.5 cm), MAM (MUAC 11.5 – < 12.5 cm) and SAM (MUAC < 11.5 cm)

Analysis of primary end point

Differences between ACT+SLDPQ vs. ACT+Placebo with respect to the primary endpoint of profound or severe anaemia with severity features in the G6PD deficient patients.

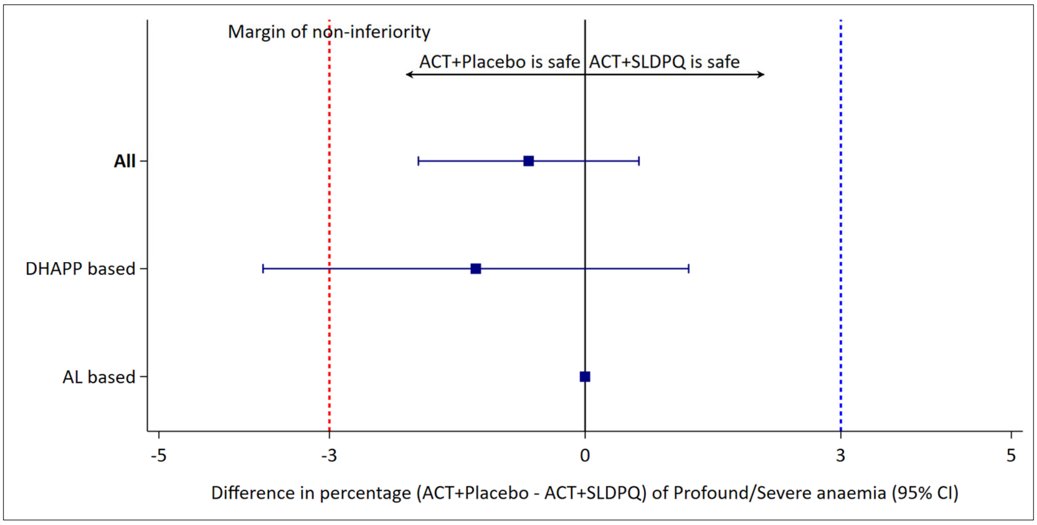

The absolute and fractional falls in haemoglobin from baseline to the nadir haemoglobin ≤ 14 days of follow up.

	ACT+SLDPQ	ACT + Placebo	p-value
**G6PDd group**	**(N=151)**	**(N=133)**	
Baseline Hb concentration (g/dL)*	10.2 (1.5)	10.4 (1.6)	0.21
Nadir Hb concentration (g/dL)*	8.9 (1.4)	9.2 (1.6)	0.095
Nadir Hb absolute change (g/dL)†	-1.1 (-4.7 – 0)	-0.9 (-4.4 – 0)	0.074
Nadir Hb fractional change (%)†	-11.1 (-36.7 – 0)	-9.0 (-40.4 – 0)	0.048
Nadir Hb fractional fall ≥ 30%‡	4 (2.7)	6 (4.5)	0.40
**G6PD heterozygous females**	**(N=56)**	**(N=63)**	
Baseline Hb concentration (g/dL)*	10.7 (1.8)	10.3 (1.6)	0.26
Nadir Hb concentration (g/dL)*	9.2 (1.4)	9.0 (1.5)	0.30
Nadir Hb absolute change (g/dL)†	-1.3 (-5.0 – 0)	-1.3 (-4.0 – 0)	1.0
Nadir Hb fractional change (%)†	-13.4 (-33.8 – 0)	-12.0 (-37.0 – 0)	0.82
Nadir Hb fractional fall ≥ 30%‡	3 (5.4)	2 (3.2)	0.55
**G6PD normal group**	**(N=350)**	**(N=367)**	
Baseline Hb concentration (g/dL)*	10.8 (1.6)	10.7 (1.6)	0.41
Nadir Hb concentration (g/dL)*	9.4 (1.6)	9.3 (1.5)	0.27
Nadir Hb absolute change (g/dL)†	-1.3 (-6.6 – 0)	-1.3 (-7.1 – 0)	0.77
Nadir Hb fractional change (%)†	-12.0 (-57.9 – 0)	-11.9 (-49.0 – 0)	0.69
Nadir Hb fractional fall ≥ 30%‡	13 (3.7)	17 (4.6)	0.54

* mean (SD)

† median (range)

‡ n (%)

Factors examined for an independent association with haemoglobin fractional fall from baseline to nadir ≤ 14 days.

Factors	Univariate model		Multivariable model	
	Slope (95% CI)	p-value	Adjusted slope (95% CI)	p-value
Age (years)	-0.43 (-0.59, -0.27)	<0.0001	-0.36 (-0.52, -0.19)	<0.0001
Sickle cell trait/disease	-2.02 (-3.41, -0.62)	0.0047	-2.01 (-3.42, -0.60)	0.0052
Splenomegaly	-2.81 (-3.97, -1.65)	<0.0001	-2.43 (-3.57, -1.29)	<0.0001
Pf asexual parasitaemia (/uL, log scale)	1.00 (0.82, 1.19)	<0.0001	0.98 (0.79, 1.16)	<0.0001
Length of illness (days)	0.69 (0.27, 1.11)	0.0013	0.72 (0.29, 1.16)	0.0011
G6PD status				
G6PD normal	Reference		Reference	
G6PDd males and females	-1.19 (-2.35, -0.020)	0.046	-0.17 (-1.32, 0.99)	0.77
G6PDd heterozygous females	0.16 (-1.49, 1.80)	0.85	-0.49 (-2.14, 1.16)	0.56
α-thalassaemia status				
Normal haemoglobin type	Reference		Reference	
Thalassemia trait (-α/-α)	-2.17 (-3.92, -0.41)	0.016	-1.36 (-3.07, 0.35)	0.12
Silent carrier (-α/αα)	-1.03 (-2.08, 0.021)	0.055	-0.57 (-1.60, 0.47)	0.28
Mg/kg dose of primaquine	-7.24 (-16.77, 2.29)	0.14	-0.51 (-10.2, 9.2)	0.92
Sex (male vs female)	0.12 (-0.89, 1.13)	0.821	-	-
Hepatomegaly	-0.73 (-3.03, 1.56)	0.53	-	-
Moderate/severe acute malnutrition	-1.40 (-7.35, 4.55)	0.64	-	-
Treatment				
AL+SLDPQ (vs AL +Placebo)	-0.18 (-1.59, 1.22)	0.798	-	-
DHAPP+SLDPQ (vs DHAPP+Placebo)	0.35 (-1.06, 1.76)	0.627	-	-

Factors not significant in the univariate model were excluded from the multivariate model except for mg/kg dose of primaquine.

Mean reticulocyte counts over time for all patients.

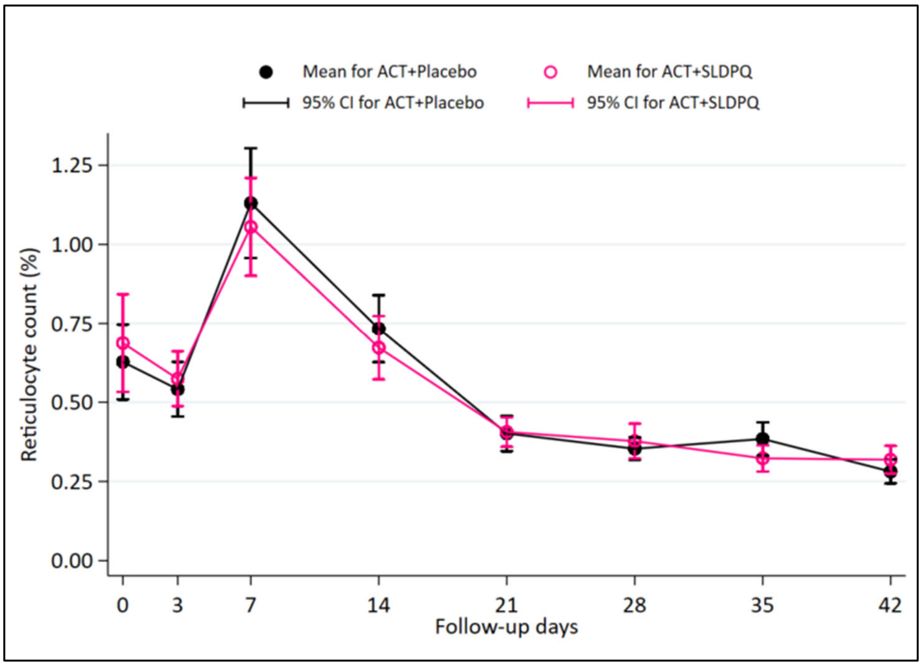

Changes in methaemoglobin over time for all patients combined in the single low dose primaquine and placebo arms.

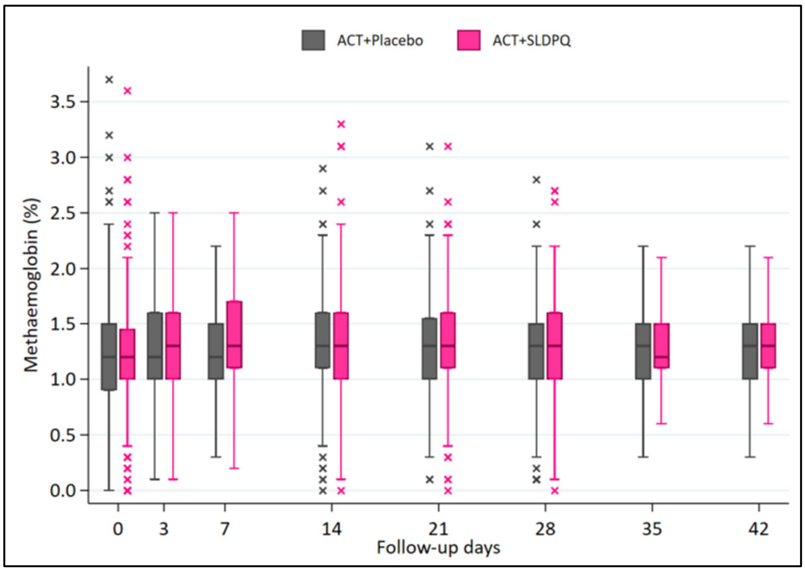

The G6PD normal patient with a methaemoglobin of 7.2% is omitted for greater clarity.

Changes in methaemoglobin concentration over time by G6PD status and primaquine allocation.

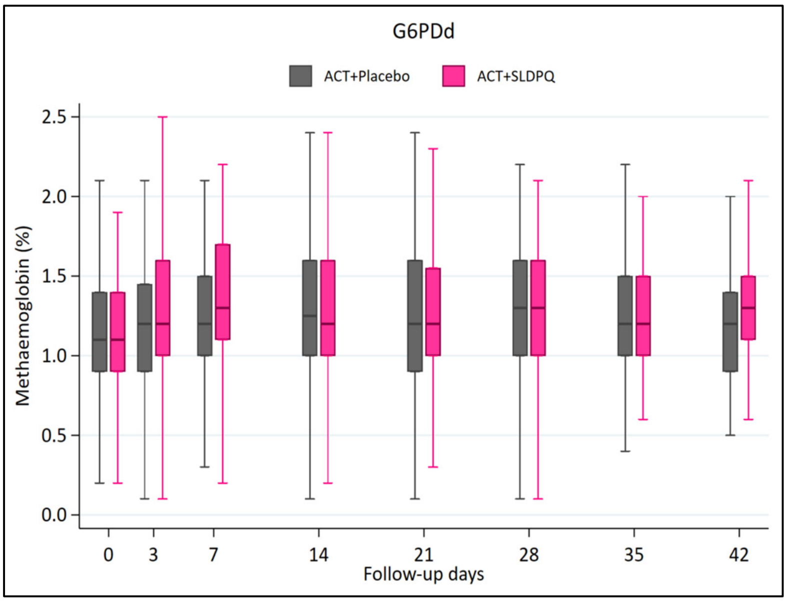

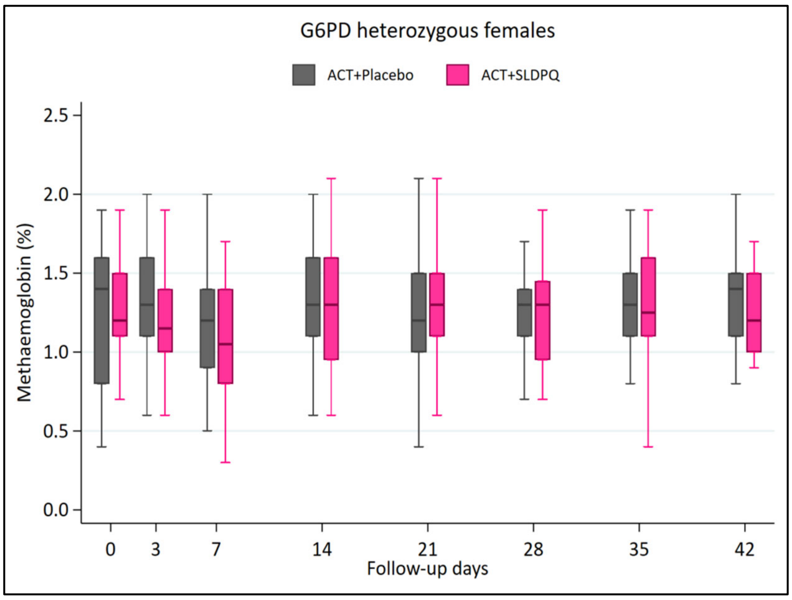

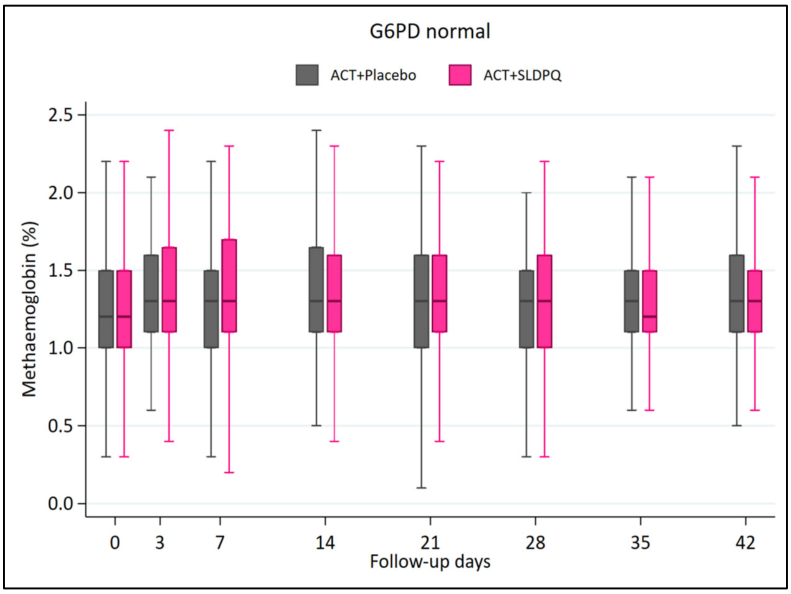
G6PD normal
ACT+Placebo
ACT+SLDPQ
Methaemoglobin (%)
2.5
2.0
1.5
1.0
0.5
0.0
0
3
7
14
21
28
35
42
Follow-up days

AST concentrations overtime in all patients.

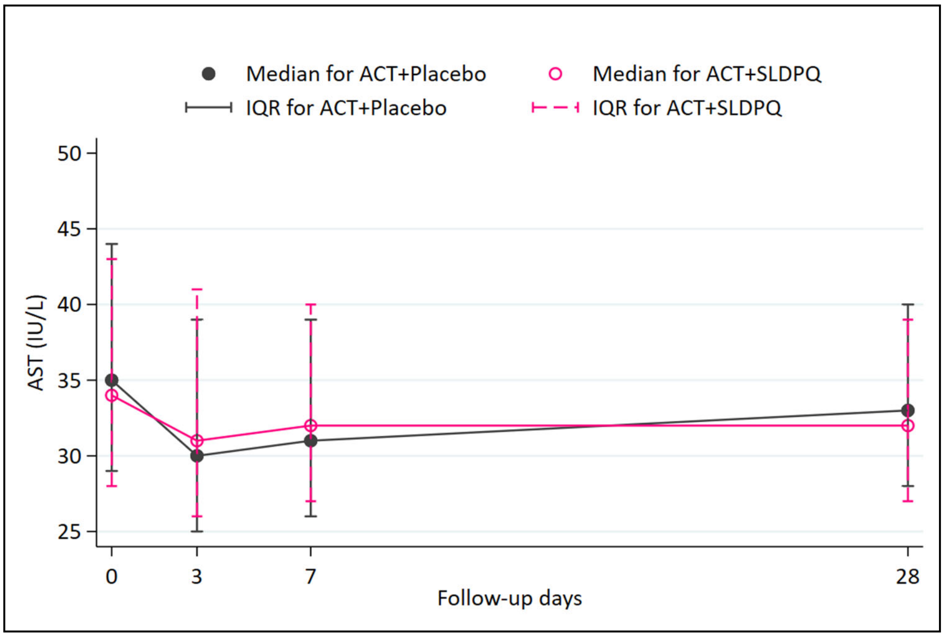

NB. N=413,408,397,392 (SLDPQ) and N=418,417,396,402 (Placebo) at day 0,3,7,28, respectively

ALT concentrations overtime in all patients.

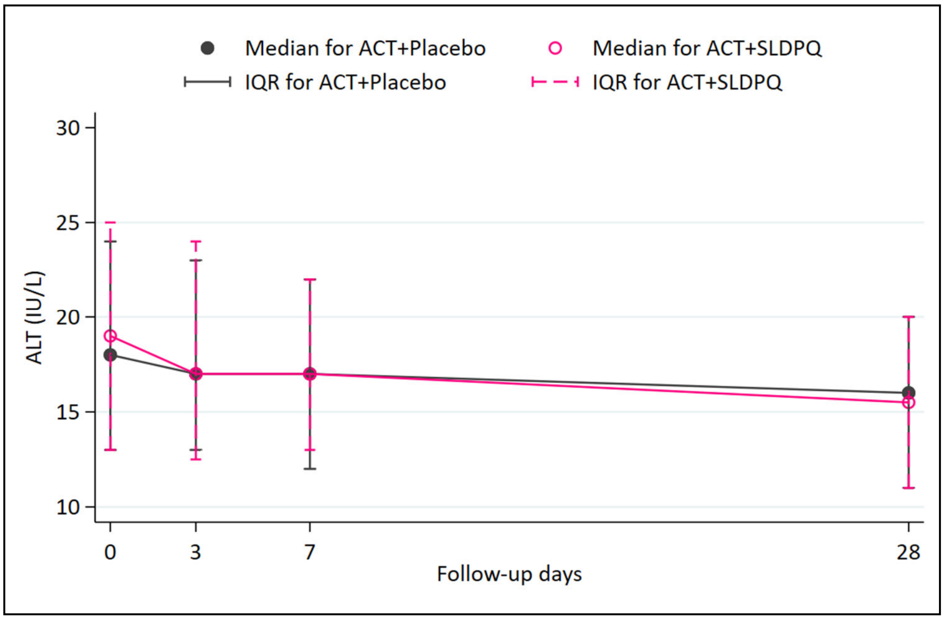

NB. N=413,408,397,392 (SLDPQ) and N=418,417,396,402 (Placebo) at day 0,3,7,28, respectively

Total bilirubin concentrations overtime in all patients.

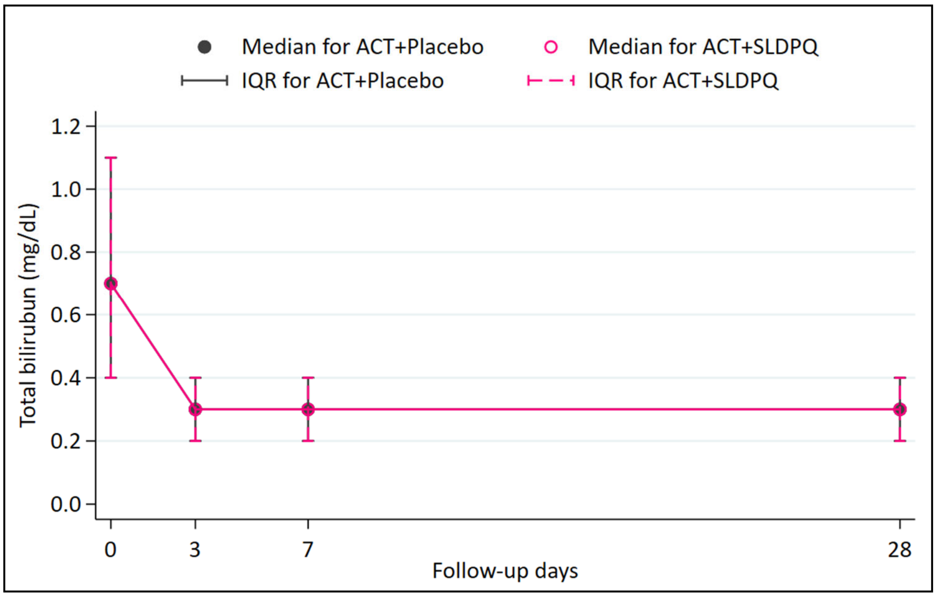

NB. N=393,378,369,353 (SLDPQ) and N=403,375,372,359 (Placebo) at day 0,3,7,28, respectively

Creatinine concentrations overtime in all patients.

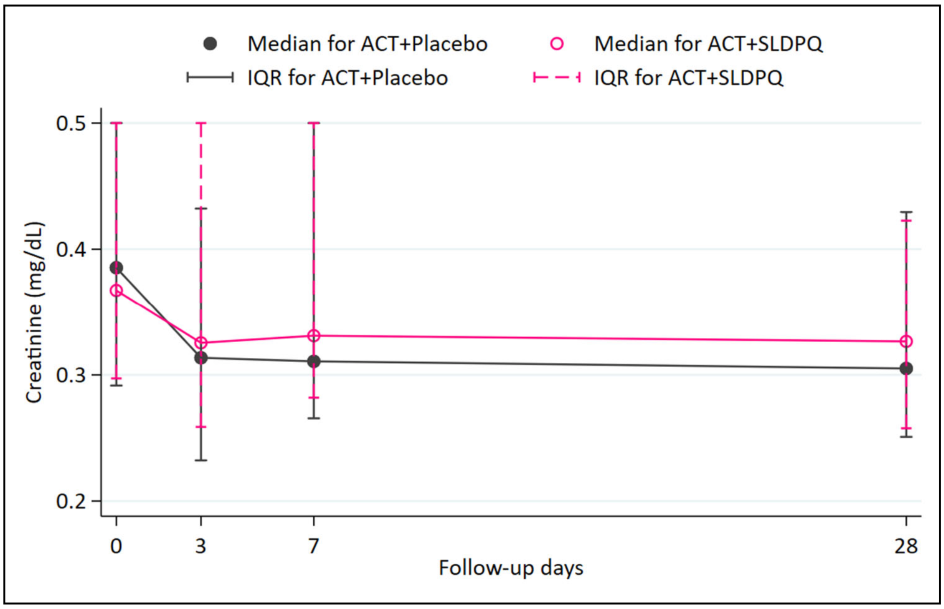

NB. N=375,369,356,343 (SLDPQ) and N=380,376,352,347 (Placebo) at day 0,3,7,28 respectively

Details of patients who were transfused. None had sickle cell trait or disease.

Country	Enlarged spleen	D0 parasite ct (/uL)	G6PD genotype	Hb type	Arm	ACT	Day transfused	D0 Hb (g/dL)	Hb transfused (g/dL)	Parasite ct transfused (/uL)	Signs of severe malaria
Uganda	N	0	DEF	HbAA	SLDPQ	DHAPP	D0	6.5	4.8	0	severe anaemia (Hb4-5) only
Uganda	Y	0	NOR	−α/αα	Placebo	AL	D0	6.4	4.9	0	severe anaemia (Hb4-5) only
Uganda	N	28,786	NOR	−α/αα	SLDPQ	DHAPP	D1	11.4	4.8	98	prostration & severe anaemia
DRC	N	660,970	DEF	HbAA	Placebo	AL	D1	8.3	5.9	586,678	Unrousable coma
Uganda	N	250,536	NOR	HbAA	SLDPQ	DHAPP	D2	8	4.6	0	None
Uganda	Y	579,443	NOR	−α/αα	SLDPQ	AL	D2	6.8	4.8	203	None
Uganda	Y	211,430	NOR	HbAA	Placebo	AL	D2	9.4	4.9	0	None
DRC	N	260	DEF	HbAA	SLDPQ	AL	D4	6.5	5.3*	0*	prostration & severe anaemia
Uganda	Y	497,818	DEF	HbAA	Placebo	AL	D4	6.5	6.5	0	None
Uganda	Y	5,860	NOR	−α/αα	Placebo	DHAPP	D5	9.8	6.7	0	None
Uganda	N	219,637	NOR	−α/αα	SLDPQ	DHAPP	D31	13.3	9.6†	1,908†	prostration, severe anaemia, haemoglobinuria

Footnote: WHO transfusion guidelines recommend transfusion for children with Hb < 4g/dl or Hb4-5g/dl with severity signs

Abbreviations: Hb - haemoglobin), ACT - artemisinin based combination treatment, D - day, N - no, Y- yes, DEF - deficient, NOR - normal, DHAPP - dihydroartemisinin piperaquine, AL - artemether lumefantrine, −α/αα - heterozygous α+ thalassaemia (silent carrier).

* latest data - 1 day prior to transfusion

† latest data - 4 days prior to transfusion

Gametocyte carriage in children treated with artemether lumefantrine.

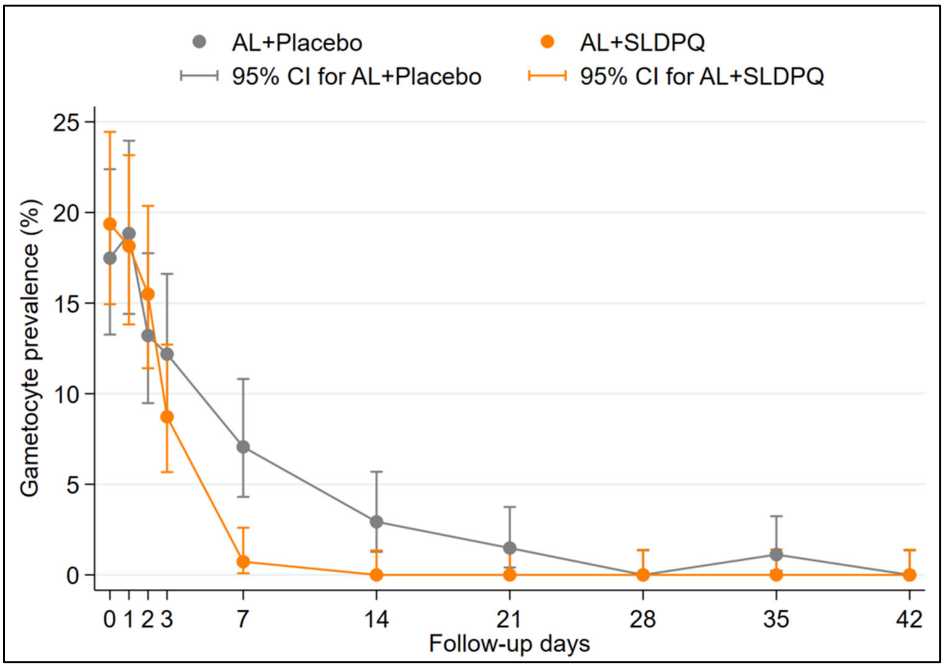

Gametocyte carriage in children treated with dihydroartemisinin piperaquine.

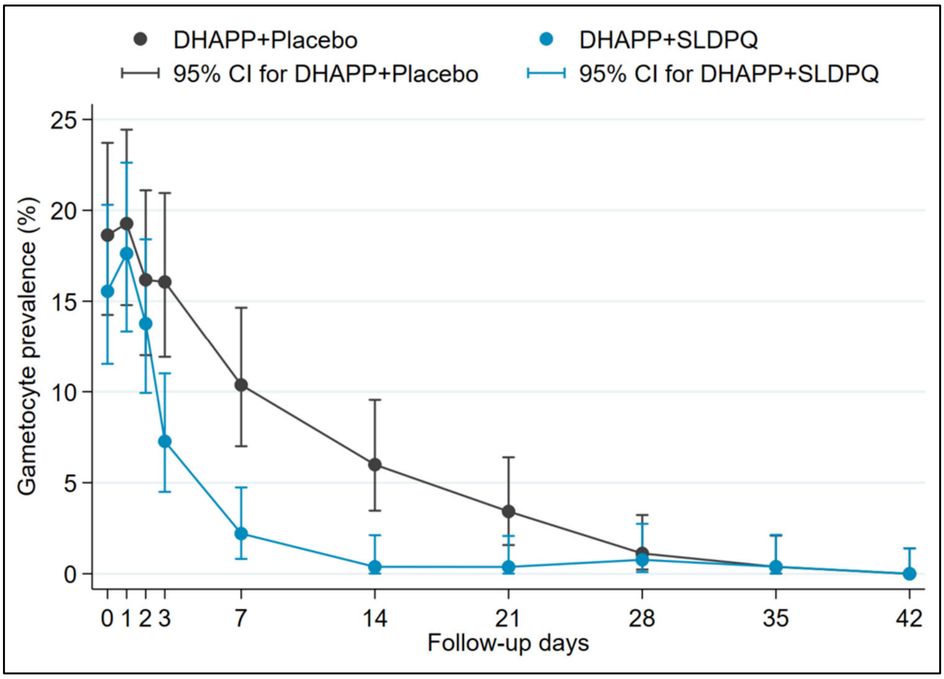

Supplement: Supplementary appendix [file EMS209522-supplement-Supplementary_appendix.pdf]
